# Phase‐Resolved Dual Control of Phenol Photodissociation at the Air–Water Interface From Structure‐Resolved Statistics

**DOI:** 10.1002/advs.76249

**Published:** 2026-06-22

**Authors:** Qiang Yin, Jialing Shi, Jinping Zhao, Chengjun Li, Yongbo Xie, Benkun Tan, Da Wang, Ziyun Wang, Yu Mao

**Affiliations:** ^1^ School of Materials and Energy Central South University of Forestry and Technology Changsha China; ^2^ College of Landscape Architecture Central South University of Forestry and Technology Changsha China; ^3^ Testing Technology Company of Changsha Research Institute of Mining and Metallurgy Co., Ltd Changsha China; ^4^ School of Civil and Architecture Engineering Hunan University of Arts and Science Changde China; ^5^ School of Chemical Sciences University of Auckland Auckland New Zealand

**Keywords:** air–water interface, electron stabilization, multiphase modeling, phenol, πσ*‐related photochemistry

## Abstract

Phenolic photodissociation at the air–water interface proceeds orders of magnitude faster than in bulk water, yet the structural origins of this acceleration remain insufficiently understood. Here, we present a descriptor‐level analysis supporting a dual‐control picture, in which phase‐dependent photodissociation reflects both πσ*‐related dark‐state accessibility and the local solvent's capacity to accommodate transferred electron density. We construct a structure‐resolved, statistics‐driven framework that bypasses snapshot‐level multireference conical‐intersection searches by identifying solvent‐side dark‐state acceptor orbitals {σ_p_*}, constructing their energy distribution ε(σ_p_*), and linking it to local microenvironment descriptors that quantify coordination saturation and directional constraint. Truncated cluster models prove unreliable because boundary microstates dominate acceptor selection and mask the intrinsic interface–bulk contrast. Periodic slab models remove this bias: the interfacial ε(σ_p_*) distribution is shifted lower by approximately 0.7 eV and substantially broadened relative to bulk, predominantly through within‐motif energy‐window shifts rather than differences in hydrogen‐bond topology. Low‐coordination, weakly constrained microenvironments correlate systematically with lower ε(σ_p_*), and small‐system SA‐CASSCF diagnostics support the same trend direction. Together, these descriptor‐level signatures indicate that the air–water interface favors both dark‐state access and transferred‐electron stabilization, providing transferable inputs for multiphase photochemical modeling and strategies for tuning interfacial reactivity through control of defect‐rich microstate supply.

## Graphical abstract

Faster phenol photolysis at the air–water interface arises from two cooperative factors: a more favorable initial microenvironment for solvent‐side electron stabilization, which lowers CI access, and a more labile hydrogen‐bond network, which more readily reorganizes to stabilize the dark‐state intermediate.

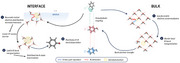

## 1 Introduction

The air–water interface functions as a reactive microreactor in many multiphase environments [1, 2], from atmospheric aerosol processing to thin‐film and foam‐based photoreactors [3, 4]. In these environments, interfacial solvation heterogeneity [5] and hydrogen‐bond network reorganizability can substantially amplify surface reactivity [6, 7]. From the standpoint of engineering and multiphase modeling, the key requirement is often not a single fully resolved mechanistic pathway but rather a set of statistically transferable, structure‐resolved descriptors that quantify how interface and bulk differ in the supply of relevant microstates and the associated energetic costs.

At the molecular level, interfacial water is more under‐coordinated, more orientationally anisotropic, and more electrostatically heterogeneous than bulk water, with a higher prevalence of hydrogen‐bond defects and dangling motifs [8, 9, 10]. These features reshape local solvation and alter the coupling between solvent structure and excited‐state reaction coordinates, so both kinetics and branching can deviate markedly from bulk behavior [11, 12]. Phenolic compounds offer a particularly instructive platform for examining this type of solvent‐modulated photochemistry. Interface‐selective ultrafast vibrational spectroscopy, for example, has shown that phenol photodissociation at the air–water interface can proceed about 10^4^ times faster than in bulk water at the same photon energy [13], suggesting that the interfacial ensemble contains a significant population of highly reactive microenvironments that promote charge separation. Understanding this amplification at the descriptor level requires examining both the kinetic and thermodynamic factors that differ between the two phases.

Most theoretical discussions of this system have emphasized kinetic accessibility: interfacial solvation defects reshape the relative energetics and coupling between the bright ^1^ππ* state and the dark‐state‐related ^1^πσ* [14], lowering the effective barrier for population transfer from the Franck–Condon region toward the bright–dark interaction regime, including crossing regions associated with conical intersections (CI) [15]. This perspective is clearly important, but it does not by itself capture the full phase dependence of reactivity. Product yield and persistence may depend not only on how readily dark or crossing regions are reached, but also on how efficiently the surrounding solvent can stabilize the ensuing charge‐separated state, especially its excess‐electron component [16]. In phenol photodissociation and related charge‐separation chemistry, the products can include phenoxy radicals, hydrated protons or proton‐transfer networks, and hydrated electrons. Among them, hydrated‐electron formation and stabilization are especially sensitive to local solvation topology: excess electrons preferentially occupy defect‐rich, dangling‐OH, or otherwise reorganizable cavity‐like acceptor environments [17, 18]. Phase differences in reactivity may therefore emerge in two coupled ways: through the tendency to access dark‐state pathways and through the energetic cost of stabilizing the products formed thereafter.

Evaluating both ways on an equal footing poses a practical challenge. A meaningful comparison between interface and bulk requires ensembles that are both structurally realistic and statistically representative. The obstacle is not simply the computational cost of multireference treatments; it is also the difficulty of preserving phase representativeness while retaining interpretable structure–energy relationships across many snapshots. Without statistical representativeness, even a formally accurate electronic‐structure method may yield a phase comparison of limited physical significance.

To address this gap, we develop a structure‐resolved, statistics‐driven framework for phase‐resolved comparison between interfacial and bulk ensembles. The framework considers two coupled controls. The first is kinetic accessibility, represented by the Franck–Condon‐region separation between the ππ* and πσ* families as an indicator of the tendency of a given microenvironment to evolve toward dark/crossing behavior. The second is stabilization propensity, represented by the energy distribution of solvent acceptor configurations that can accommodate excess‐electron density, thereby linking electron‐stabilization cost to local solvation topology around the solvent‐side electron‐accumulation region (SEAR), as a trend‐level descriptor rather than a direct measure of product stabilization energy or reaction rate. Small‐system SA‐CASSCF calculations provide independent and qualitative support, confirming that local solvent perturbations can directly reshape acceptor energetics and the associated crossing trends. On this basis, we use snapshot‐level structure–energy relationships and phase‐specific microstate supply to assess how interface and bulk differ in their capacity to generate and sustain low‐energy acceptor environments favorable for electron stabilization. The resulting descriptor‐level statistics provide transferable inputs for incorporating interfacial effects into multiphase photochemical models and suggest experimentally accessible routes for tuning interfacial reactivity.

## 2 Computational Methods

### 2.1 System Preparation, Classical MD, and Cluster Extraction

Interface and bulk ensembles were generated by classical molecular dynamics simulations using systems containing one phenol molecule and approximately 2.1 × 10^3^ water molecules. The bulk model used a ca. 40 Å cubic box, while the interfacial model was obtained by adding ca. 30 Å vacuum along the surface normal of an equilibrated phenol–water slab. Water was described with the rigid four‐site OPC model [19], and phenol was parameterized with GAFF [20] using RESP charges [21] derived in Gaussian 16 [22]. Each phase was equilibrated for approximately 20 ns, during which about 20 000 trajectory frames were collected for analysis. From these trajectories, 30 snapshots were selected at equal time intervals for each phase so that the extracted geometries would remain statistically representative for subsequent electronic‐structure calculations.

Trajectory‐consistent cluster models were obtained by extracting phenol together with surrounding water molecules within a 10.0 Å radius of the phenol center of mass. Minimum‐image reconstruction was applied under periodic boundary conditions to preserve geometric continuity and hydrogen‐bond connectivity. No geometry optimization was performed after extraction. Instead, the MD geometries were used directly for single‐point calculations and wavefunction analysis so that post‐processing relaxation would not distort the sampled microenvironments.

### 2.2 Cluster Electronic‐Structure Calculations and Wavefunction Analysis

All cluster calculations were carried out in Gaussian 16. Ground‐state single‐point calculations employed B3LYP/def2‐TZVP [23, 24] to obtain molecular orbitals and wavefunction information. Excited states were then computed at the same level with TDDFT [25] using NStates = 50, yielding vertical excitation energies together with the donor and acceptor orbital energies relevant to the excitation families of interest. These data were used to establish linear trend mappings between excitation energies and donor–acceptor orbital gaps and to verify the statistical stability of excitation character near the FC region through NTO diagnostics.

Partial densities of states (PDOS) and O─H‐stretch‐induced density differences, Δρ, were evaluated in Multiwfn [26, 27] from the Gaussian wavefunction outputs. PDOS analysis was used to screen virtual orbitals and define the solvent‐stabilized dark‐state acceptor manifold {σ_p_*}. The density‐difference analysis, in turn, was used to localize the region relevant to initial electron accumulation, which then served as the spatial reference for the microenvironment descriptors introduced below (see Section S3.3).

### 2.3 Periodic Configuration Generation and Single‐Point Calculations

To avoid potential boundary effects associated with finite cluster models, all phase‐resolved statistics were ultimately based on periodic models extracted from CP2K ab initio molecular dynamics trajectories. The interfacial model contained one phenol molecule and ca. 230 water molecules in a 24 × 24 × 12Å^3^ water slab, with ca. 15 Å vacuum on both sides along the surface normal, whereas the bulk model contained one phenol molecule and ca. 590 water molecules in a 26 × 26 × 26Å^3^ periodic cell. AIMD simulations were performed at 298.15 K with a 0.5 fs time step using a CSVR thermostat; the interfacial system was propagated for 10 000 NVT steps, while the bulk system used 3000 NVT steps followed by 7000 NPT steps. The CP2K MGRID cutoff and REL_CUTOFF were 350 and 35 Ry, respectively, with $\mathrm{EPS}\_\mathrm{SCF}\;=1\times 10^{-5}$ and Broyden mixing. From the last 3000 frames of each trajectory, 20 statistically separated snapshots were selected for periodic single‐point calculations and PDOS/Δρ analysis. The single‐point calculations were performed in CP2K [28] at the PBE0/TZV2P‐MOLOPT‐GTH(q4) level [29] and accelerated by ADMM using AUX_FIT admm‐dzp‐q4 [30]. This setup provided orbital energies and wavefunction information for subsequent analysis.

To ensure consistency across both phases and all snapshots, orbital energies were referenced to the phenol HOMO π orbital, ε(π), which was taken as the common zero for gap construction and statistical comparison. The resulting orbital and wavefunction information was further processed in Multiwfn under the same protocol used for the cluster models, yielding ε(σ_p_*), SEAR localization, and the inputs needed for structure–energy correlation analysis.

### 2.4 SA‐CASSCF Diagnostics and Scan‐Based Crossing‐Region Indicators

To assess whether the structure–energy relationships identified by the framework are consistent with crossing‐region trends, we carried out state‐averaged CASSCF calculations in Gaussian 16. The calculations used SA‐CASSCF(8,8)/6‐31+G(d,p) [31], with an active space including the phenyl π/π* orbitals and the σ/σ* pair of the phenolic O─H bond (Figure S4). Initial orbitals were taken from MP2/6‐31+G(d,p) [32], and ten singlet roots were included in the state averaging. Microenvironment tests were performed on phenol and phenol–water clusters by systematically perturbing the configuration of the water molecule hydrogen‐bonded to the phenolic O─H group.

In addition, rigid scans were carried out along the phenolic O─H bond to obtain operational indicators for approaching the interaction region. Starting from r(O─H) = 0.98 Å, the bond length was increased in 0.05 Å increments over 15 points. Ten singlet roots were computed at each point, and state identity was tracked using dominant excitation or configuration character. Intersections or near‐degeneracies along the scan were used as working markers: the S_2_/S_1_ crossing was denoted CI1 and the S_2_/S_0_ crossing CI2. These were treated as scan‐based indicators rather than minimum‐energy CI optimizations. The effective crossing‐barrier indicator was defined as the energy difference between CI1 and the FC reference point, and this quantity was later compared with ΔE_FC_ at the trend level. Additional computational details and all numerical settings are given in the Supporting Information.

## 3 Framework Design

**FIGURE 1:**
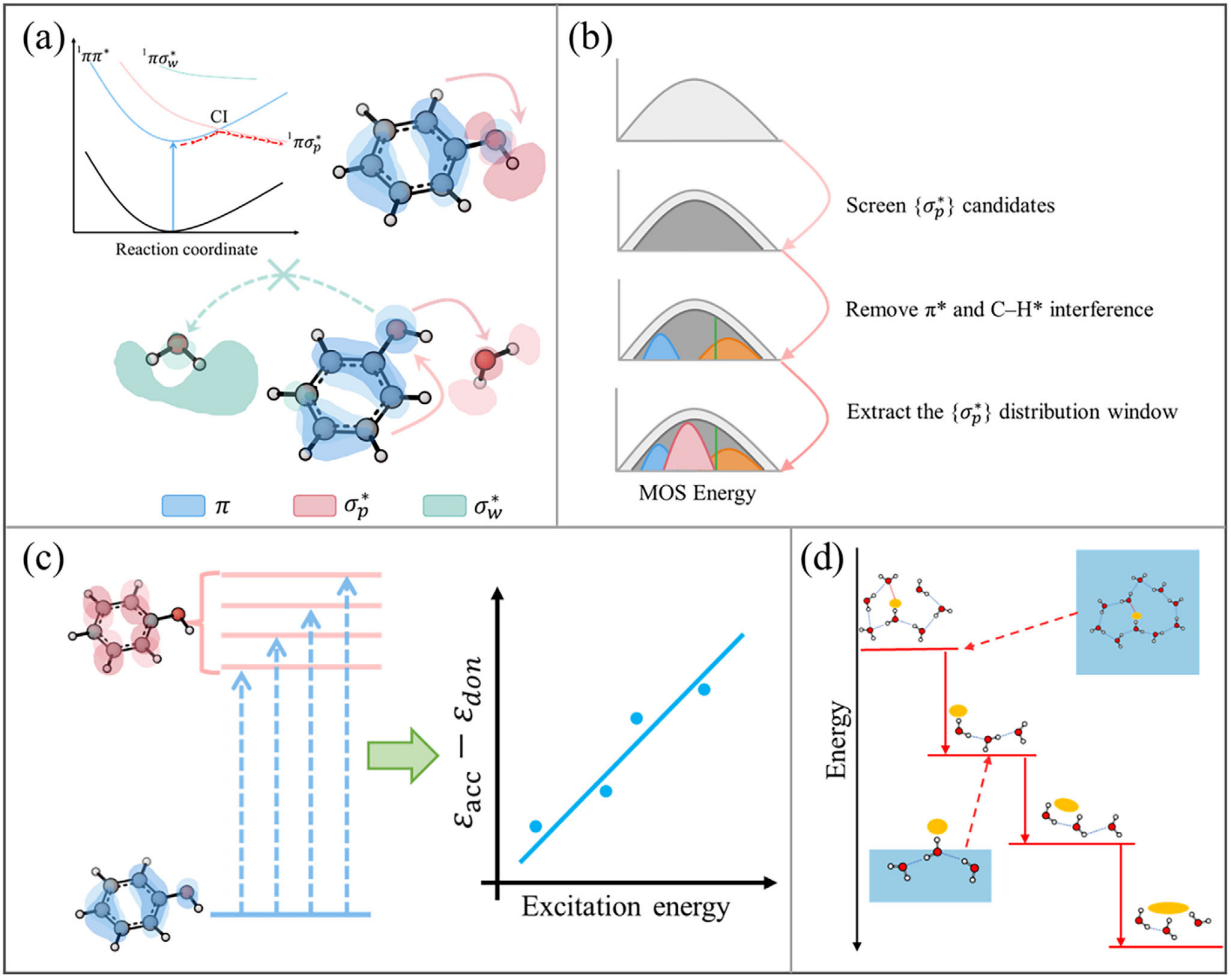
Conceptual basis and core workflow of the present framework. (a) Schematic illustration of phenol photodissociation after photoexcitation, comparing isolated phenol and locally hydrated phenol, and highlighting why solvent‐side dark‐state acceptor orbitals must be identified. (b) Module 1: PDOS‐based screening of the solvent‐stabilized dark‐state acceptor manifold, {σ_p_*}. Light gray: all virtual orbitals; dark gray: preliminarily screened candidates; blue: major π* region; brown: major C–H* region; pink: final {σ_p_*} distribution; green line: upper bound of the orbital‐energy window. (c) Module 2: Mapping donor–acceptor orbital gaps onto excitation energies of the relevant excited‐state families to establish fitted gap–energy relationships. (d) Module 3: After identifying the SEAR (yellow region), its surrounding microsolvation environment is linked to the corresponding energetic quantities, enabling phase‐dependent comparison of product stabilization.

As discussed above, snapshot‐level multireference exploration of excited‐state surfaces is computationally prohibitive for statistically representative ensembles. The framework therefore extracts the key information needed for phase comparison through three linked modules, followed by a phase‐level integration step (Figure 1).

Module 1 identifies the solvent‐stabilized dark‐state acceptor manifold, {σ_p_*}, from the virtual‐orbital space and constructs its acceptor‐level energy distribution, ε(σ_p_*), as a trend‐level descriptor of the relative energetic cost for solvent‐side electron accommodation. Module 2 maps donor–acceptor orbital gaps onto the vertical excitation energies of the relevant ^1^πσ_p_* and ^1^ππ* families and uses their Franck–Condon‐region separation, ΔE_FC_, as a trend‐level indicator of CI‐related accessibility. Module 3 uses O─H‐stretch‐induced density differences, Δρ, to localize the SEAR associated with each acceptor state and then quantifies the surrounding microsolvation environment through the SEAR‐centered descriptors N and P. In the final integration step, these structure–energy relationships are combined with phase‐specific microstate supply to compare how interfacial and bulk environments differ in descriptor‐level tendencies toward low‐energy electron accommodation and subsequent product stabilization.

Module 1. Solvent‐stabilized dark‐state acceptor set {σ_p_*} and ε(σ_p_*).

Upon photoexcitation of isolated phenol, the system evolves primarily within the phenol‐centered excited‐state manifold: the bright ππ* state couples nonadiabatically to the connected πσ* dark state, which in turn drives O─H bond dissociation. In this limit, the dark‐state acceptor character is mainly intrinsic to phenol itself, and no additional solvent‐side acceptor states need to be introduced (Figure 1a). This picture changes once the phenolic hydroxyl is embedded in a local hydrogen‐bond network with surrounding water molecules. The electron density associated with the dark‐state acceptor channel no longer needs to remain localized on the intrinsic phenolic σ_OH_* character, but may instead be redistributed through the hydrogen‐bond network into O─H antibonding orbitals of nearby solvent molecules [33]. In other words, only solvent molecules with suitable local hydrogen‐bond connectivity can serve as effective acceptors for the transferred electron density. Identifying these solvent‐side dark‐state acceptor orbitals is therefore the necessary first step in the explicit‐solvent case. The spatial localization and energy of these solvent‐side acceptor states are expected to influence where the electron is accommodated after transfer and how readily the resulting charge‐separated product state can be stabilized. The first task is therefore to identify, from the manifold of virtual orbitals, those states that can act as solvent‐side acceptors for electron density transferred out of the phenol‐centered dark state while retaining pronounced σ_OH_*‐like character relevant to that dark‐state acceptor channel. These orbitals define the solvent‐stabilized dark‐state acceptor manifold, {σ_p_*}. Their energies collectively give the phase‐resolved acceptor‐level distribution ε(σ_p_*), which is taken here as an indicator for stabilization cost.

The selection is based on a direct physical criterion: orbitals that inherit more of the acceptor character relevant to the phenolic dark state should be more effective destinations for the transferred electron density. We therefore use PDOS analysis to quantify this character in each virtual orbital and implement the selection through a broad‐capture, tiered‐filtering scheme (Figure 1b). In the broad‐capture stage, virtual orbitals are ranked by PDOS‐OH–H, the projection on the phenolic hydroxyl H, and only the top *k* candidates are retained because this projection most directly tracks the dark‐state acceptor character. To suppress π‐type false positives, we then screen against PDOS‐ring–C. Aromatic π* orbitals are not the target acceptor states; however, they can still exhibit appreciable PDOS‐OH–H because they may contain mixed contributions from phenolic O─H antibonding character. Their dominant real‐space signature, however, remains concentrated on the aromatic ring and can therefore be removed through the ring‐C projection. In the final stage, an upper cutoff E_cut_ is imposed to exclude levels dominated by C–H* character and by higher‐energy solvent or diffuse mixing. E_cut_ is determined statistically from the cumulative PDOS‐ring–H distribution using a 10% cumulative‐area criterion in order to retain a controlled fraction of the high‐energy {σ_p_*} tail, so that the resulting trends can be examined for directional consistency rather than being defined solely by the low‐energy portion. The remaining orbitals are assigned to {σ_p_*} for each snapshot. Pooling them over all snapshots yields ε(σ_p_*), whose phase‐resolved distribution reflects both the energetic cost of electron accommodation and the statistical prevalence of solvent‐stabilized acceptor configurations. More detailed definitions of the PDOS fragments, computational settings, selection principles, and threshold choices are provided in the Supporting Information.

Module 2. FC‐region excitation mapping and ΔE_FC_.

Module 1 provides single‐orbital energies; for phase comparison, these must be converted into excitation energetics of the corresponding excited‐state families. The purpose of this mapping is to establish a consistent descriptor for comparing excitation‐energy trends across many snapshots, not to reconstruct full excited‐state potential‐energy surfaces. Accordingly, the resulting ΔE_FC_ should be interpreted as a Franck–Condon‐region accessibility indicator for πσ*‐related dark/crossing behavior. Guided by NTO diagnostics showing that the dominant donor–acceptor pattern within a given excitation family remains statistically consistent under solvent variation, we approximate family‐level vertical‐excitation trends by the corresponding donor–acceptor orbital gaps (Figure 1c).

For the cluster models used for calibration, we fit linear relationships of the form.

(1) $$E_{exc}^{\left(m\right)}\approx a^{\left(m\right)}\Delta \varepsilon^{\left(m\right)}+b^{\left(m\right)},m\in \left\{\pi \pi^{*},\pi \sigma^{*}\right\}$$

with

(2) $$\Delta \;\varepsilon^{\left(\pi \sigma^{*}\right)}=\varepsilon \left(\sigma_{p}^{*}\right)-\varepsilon \left(\pi \right),\Delta \;\varepsilon^{\left(\pi \pi^{*}\right)}=\varepsilon \left(\pi^{*}\right)-\varepsilon \left(\pi \right)$$

This mapping converts $\varepsilon (\sigma_{p}^{*})$ into the corresponding $\pi \to \sigma_{p}^{*}$ excitation‐energy distribution and thereby places the solvent‐side acceptor energetics on the same excitation‐energy scale as the competing bright‐state channel. On this basis, we define the Franck–Condon‐region indicator

(3) $$\Delta E_{\mathrm{FC}}=E_{\mathrm{exc}}^{\left(\pi \sigma^{*}\right)}-E_{exc}^{\left(\pi \pi^{*}\right)}w$$

which is used here as a trend‐level measure of the tendency of a given microenvironment to favor access to πσ*‐related dark or crossing behavior. This quantity is not intended to replace explicit CI energies or barriers. Rather, its use is motivated by the independently calibrated relationship between Δ*E*_FC_and a scan‐based CI indicator. Because the fitted slope *a* ^(*m*)^is nearly invariant within a given excitation family, Equation (3) can be further reduced to

(4) $$\Delta \;E_{FC}=a\left[\varepsilon \left(\sigma_{p}^{*}\right)-\varepsilon \left(\pi^{*}\right)\;\right]+c$$

where *a* and *c* are effective constants obtained by combining the two family‐specific linear calibrations. The calibrated $E_{\mathrm{exc}}^{\left(m\right)}--\Delta \varepsilon^{\left(m\right)}$ relationships are shown in Figure S6, and the linear relationship between Δ*E_FC_* and the scan‐based CI indicator is shown in Figure S5b.

Module 3. SEAR localization, descriptors (N, P), and phase‐resolved stabilization potential.

While Module 2 provides a trend‐level kinetic indicator for access to πσ*‐related dark or crossing behavior, phase comparison also requires a thermodynamic perspective on how readily the transferred electron can be accommodated and stabilized once it enters the solvent‐side acceptor manifold. Module 3 addresses this problem in two steps: it first identifies, for each solvent‐side dark‐state acceptor orbital, the spatial region in which transferred electron density is most likely to accumulate initially, and then quantifies the local microsolvation environment around that region. This establishes the structure–energy relationships needed to assess which local solvent environments are more or less favorable for product stabilization.

This step follows the established picture of aromatic‐alcohol πσ*photochemistry, in which elongation of the phenolic O─H bond promotes transfer of electron density toward solvent‐side acceptor states [33]. For each snapshot, we therefore impose an O─H stretch perturbation of Δr_OH_ = 0.10 Å and evaluate the density difference.

(5) $$\Delta \rho \;\left(r\right)=\rho_{\mathrm{stretched}}\;\left(r\right)-\rho_{\mathrm{eq}}\left(r\right)$$

This perturbation was chosen as a compromise between response strength and orbital continuity. In the scan calculations, CI1 is typically approached only at larger O─H elongations (about 0.2 Å beyond equilibrium [33]), whereas perturbations in that regime often induce substantial state or orbital mixing and occasional orbital reordering, which would compromise robust matching of the corresponding acceptor orbital across snapshots. The chosen elongation therefore remains below the crossing region while still producing a clear density response. The dominant Δρ accumulation region is then matched to the dominant real‐space density region of the corresponding $\sigma_{p}^{*}$ orbital. This matched region is defined as the primary solvent‐side electron‐accumulation region (SEAR) for that acceptor and serves as the spatial reference for the subsequent microenvironment analysis.

Once the SEAR is identified, the transferred electron density is typically associated with one or two O─H bonds of the corresponding solvent water. For the one‐bond case, we define a conical counting volume (Figure S17) along that O─H bond vector, with a half‐angle of 60° and a radial cutoff of 4.0 Å, in order to capture the nearby microsolvation environment most directly relevant to the acceptor O─H direction while minimizing contributions from more distant, weakly directional surroundings. The rationale and sensitivity of this SEAR‐centered conical definition are further examined in Section S10.3. Two SEAR‐centered descriptors are then defined. *N* counts water oxygens accepting two hydrogen bonds and thus represents the saturated‐acceptor background. *P* quantifies nearby unsaturated acceptors around the acceptor hydrogen through a weighted directional metric and thereby captures short‐range directional pressing. Pairing $\varepsilon (\sigma_{p}^{*})$ with (*N*,*P*)across snapshots yields the structure–energy statistics used throughout this work. These relationships reveal which local solvent environments favor lower‐energy solvent‐side acceptors and are therefore more conducive to electron accommodation and subsequent stabilization. Combined with phase‐specific microstate supply and hydrogen‐bond‐network reorganizability, they provide a phase‐resolved, qualitative assessment of relative product stabilization (Figure 1d). Details of the density‐difference‐map construction, hydrogen‐bond criteria, and *P*‐value definition are provided in the Supporting Information.

## 4 Results and Discussion

### 4.1 Validation of Acceptor Identification and SEAR Localization

The present workflow rests on two operational definitions: identification of the solvent‐stabilized dark‐state acceptor manifold, {$\sigma_{p}^{*}$}, by tiered PDOS screening, and localization of the solvent‐side electron‐accumulation region (SEAR) from the spatial overlap between the O─H‐stretch‐induced density response and the density distribution of the corresponding unperturbed orbital. Before proceeding to phase‐resolved statistics, we first examine whether these two definitions are physically reasonable, i.e., whether they recover the acceptor patterns expected from the established picture of aromatic‐alcohol πσ* photochemistry.

**FIGURE 2:**
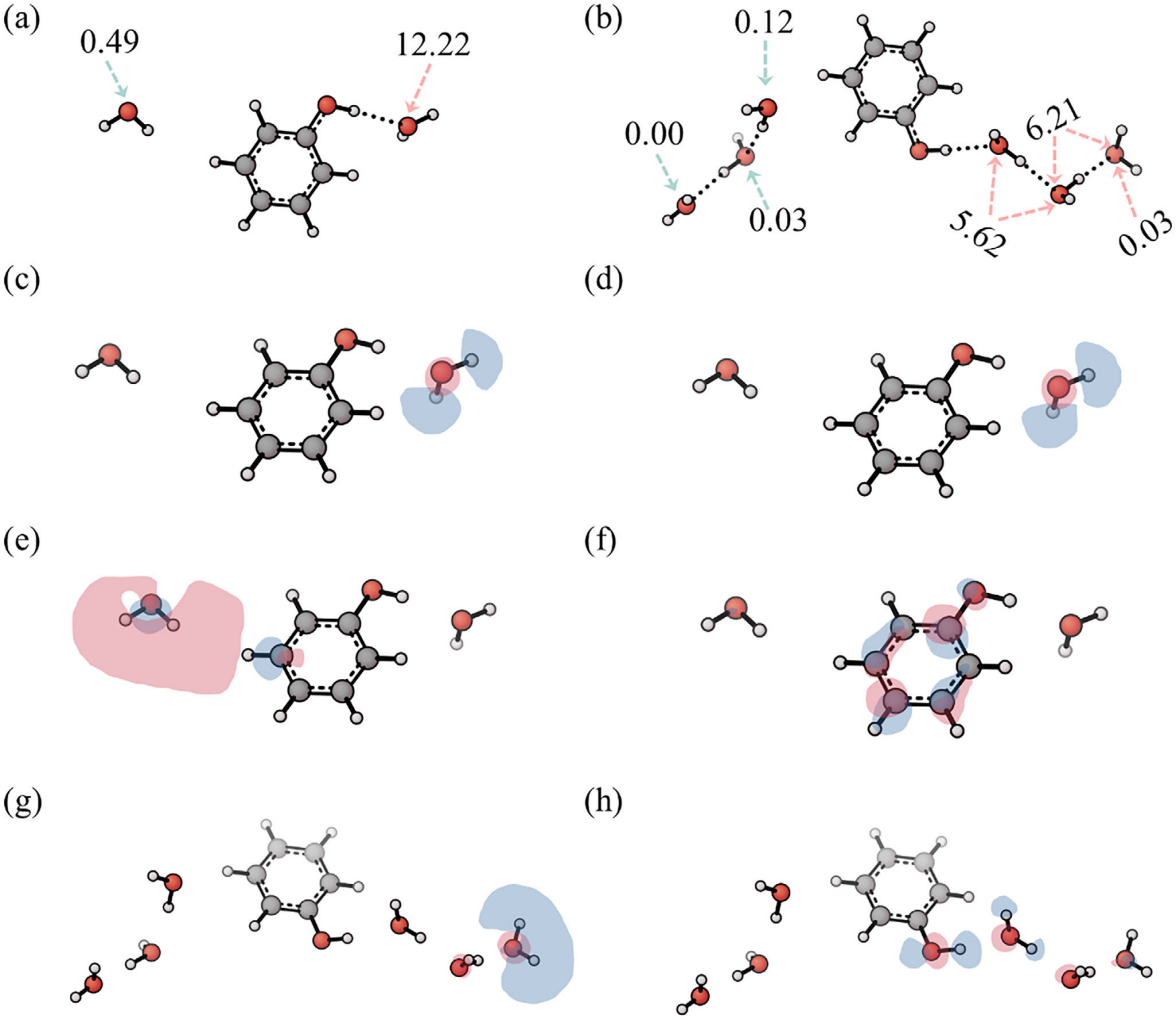
(a, b) Two representative phenol–H_2_O clusters. Pink and green arrows denote virtual orbitals mainly distributed on waters connected to and disconnected from the phenolic hydroxyl‐centered hydrogen‐bond network, respectively; the numbers are the corresponding PDOS‐OH–H values. (c, e) Orbital‐density distributions of orbitals 36 (PDOS‐OH–H = 12.22) and 38 (PDOS‐OH–H = 0.49) in structure 1. (d, f) Corresponding Δρmaps for orbitals 36 and 38 in structure 1. (g) Orbital‐density distribution of orbital 56 (PDOS‐OH–H = 0.03) in structure 2. (h) Corresponding Δρ map for orbital 56 in structure 2.

Representative configurations with visually clear asymmetry provide a direct test (Figure 2a,b): in each case, one side of the phenol is connected to a hydroxyl‐centered hydrogen‐bond network, whereas the other side is not. Under such conditions, virtual orbitals whose density is in both cases mainly distributed over waters on the hydrogen‐bond‐connected side consistently show substantially larger PDOS‐OH–H values than orbitals localized on waters outside the network. This agrees with the general physical expectation that solvent‐side acceptor orbitals relevant to the dark‐state electron‐transfer channel should preferentially reside on waters connected through the phenolic hydrogen‐bond network. At the same time, some orbitals with evident π* or C–H* character may still display non‐negligible PDOS‐OH–H values, but these false positives are effectively excluded by the subsequent PDOS‐ring–C and PDOS‐ring–H filters. The tiered PDOS protocol therefore does not merely enhance the target signal; it also suppresses spurious contributions and restricts the final selection to orbitals genuinely relevant to the solvent‐side dark‐state acceptor channel.

We next examine whether the SEAR definition captures the physically meaningful region for initial solvent‐side electron accumulation. For orbitals retained in {$\sigma_{p}^{*}$}, the dominant Δρ response induced by O─H stretching is mainly distributed on waters within the phenolic hydrogen‐bond network and exhibits clear spatial overlap with the density of the corresponding unperturbed orbital (Figure 2c,d). Within the same structure, this overlap generally becomes more pronounced as PDOS‐OH–H increases (Figures S11 and S13). By contrast, for orbitals with smaller PDOS‐OH–H, the Δρ response may still appear on phenol itself or near network waters, but it shows little or no intersection with the density distribution of the corresponding orbital (Figure 2e,f). An instructive limiting case is shown in Figure 2g,h: although the orbital density is almost entirely located on the terminal water of the network, its PDOS‐OH–H remains anomalously small because the orbital‐density region and the Δρ response region barely overlap. This case makes clear why neither Δρ alone nor the raw orbital‐density distribution alone is sufficient. The solvent‐side region relevant to initial dark‐state electron accumulation can only be defined by linking the dark‐state‐related density response to the spatial distribution of the original acceptor orbital.

Taken together, these tests show that tiered PDOS screening and overlap‐based SEAR determination are methodologically consistent and converge on the same physically plausible acceptor‐side picture: both identify solvent‐side regions accessible through the phenolic hydrogen‐bond network. This consistency justifies their use as the working definitions for the subsequent phase‐resolved statistics of ε($\sigma_{p}^{*}$) and the associated structure–energy analysis.

### 4.2 Boundary Bias and Suppressed Phase Contrast in Truncated Clusters

**FIGURE 3:**
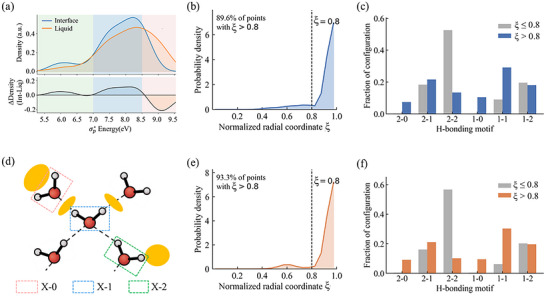
(a) Distributions of ε(σ_p_*) for the interfacial and bulk truncated‐cluster ensembles. (b, e) SEAR probability‐density maps for the interfacial (blue) and bulk (orange) phases, respectively. (c, f) Background abundances of all waters belonging to the topology‐defined motif classes in the interior and boundary regions for the interfacial and bulk truncated‐cluster ensembles, respectively. (d) Schematic illustration of the three hydrogen‐bond‐topology families of acceptor‐site waters, X‐0, X‐1, and X‐2, with X = 1 or 2. These families are defined by the hydrogen‐bond numbers of the central water molecule: the first number denotes the number of hydrogen bonds accepted by the oxygen atom, and the second the number donated by the hydrogen atoms.

The cluster extraction protocol was designed to retain as much intrinsic interface–bulk contrast as possible. To this end, multiple solvation shells were preserved, and a normalized radial coordinate, ξ, was used to separate the phase‐representative interior from the truncation‐affected boundary. Waters with ξ ≤ 0.8 were assigned to the interior, which accounts for ∼70% of all waters (see the Supporting Information for details). Even with this partitioning, however, the acceptor‐level distributions from the truncated‐cluster ensembles remain strongly overlapped. Across 30 snapshots per phase, the interfacial and bulk ε(σ_p_*) distributions differ only weakly: the interface shows only slight enrichment in the low‐energy window, and the mean shift is only ∼0.2 eV (Figure 3a). Equation (4) makes the implication direct: because ε(π*) is essentially indistinguishable between the two phases (Figure S9a), any phase dependence in ΔE_FC_ must arise almost entirely from ε(σ_p_*). If ε(σ_p_*) itself fails to separate interface and bulk, ΔE_FC_ cannot retain a recognizable phase‐contrast signal, and the cluster statistics would imply very similar barrier trends in both phases. That inference is difficult to reconcile with the strong interfacial acceleration observed experimentally. The more plausible explanation is not failure of the indicator, but suppression of the intrinsic phase signal by model bias.

To distinguish between these possibilities, we computed the initial electron‐response regions corresponding to all identified acceptor orbitals and examined their spatial distribution. The result is unambiguous: although the interior contains most waters, more than 90% of the SEARs in both phases lie in the truncation‐affected boundary region with ξ > 0.8 (Figure 3b,e). Consistently, when the background abundances of all water configurations in the clusters are analyzed according to the local hydrogen‐bond topologies illustrated in Figure 3d, the resulting motif distributions are found to be nearly identical in the two phases, indicating that the original phase specificity has been largely disrupted by the cluster model (Figure 3c,f). In truncated clusters, the initial electron response is therefore not preferentially hosted by the phase‐representative interior but is instead overwhelmingly anchored to a truncation‐induced permissive boundary environment. The intrinsic interface–bulk contrast that should appear in ε(σ_p_*) and, through it, in ΔE_FC_ is consequently suppressed by the statistical homogenization imposed by boundary‐dominated sampling.

This bias becomes clearer when the SEAR‐identified acceptor sites are examined within the same topology‐based grouping. For each family, we compare both its background abundance among all waters and its representation among SEAR‐identified acceptor sites. In the truncated clusters, acceptor selection is dominated by X‐1 motifs (∼60%), followed by X‐0 (∼30%), whereas the saturated X‐2 family is rare (Table 1). This distribution is consistent with the established preference of excess electrons for under‐coordinated, dangling‐rich environments [18, 34]. Crucially, however, the boundary bias cannot be reduced to a simple supply effect. For X‐0 motifs, the table shows a boundary‐to‐interior abundance ratio of approximately 14:1, so their dominance in the truncation‐affected region is unsurprising. The dominant X‐1 family behaves differently. Its background abundance ratio is only about 2:1 between boundary and interior, which means that if acceptor selection were controlled by motif identity alone, X‐1 acceptor sites should still be distributed substantially in both regions rather than being concentrated almost entirely at the boundary. The observed result is the opposite: X‐1 selection remains strongly exterior‐biased. What emerges here is therefore not a simple supply imbalance, but an internal selection penalty operating in the interior. Even within the same motif family, interior microenvironments are systematically less competitive than boundary ones as acceptor sites. This penalty anchors electron response to truncation‐affected boundary microstates and compresses the true interface–bulk contrast. Truncated clusters are therefore not an adequate baseline for phase‐resolved statistics intended for parameterization; periodic models are required.

**TABLE 1:** Motif‐family composition of SEAR‐identified acceptor‐site waters and boundary/interior background abundance ratios in truncated interfacial and bulk clusters.

Phase	Motif family	SEAR fraction (%)	bound/inter background ratio
Interface	X–0	23.32	14–20
Interface	X–1	66.87	1.4–2.0
Interface	X–2	6.74	∼0.3
Bulk	X–0	31.85	14–20
Bulk	X–1	56.45	1.4–2.0
Bulk	X–2	8.38	∼0.3

### 4.3 Phase‐Resolved ε(σ_p_*) Separation and Structure–Energy Relationships in Periodic Models

**FIGURE 4:**
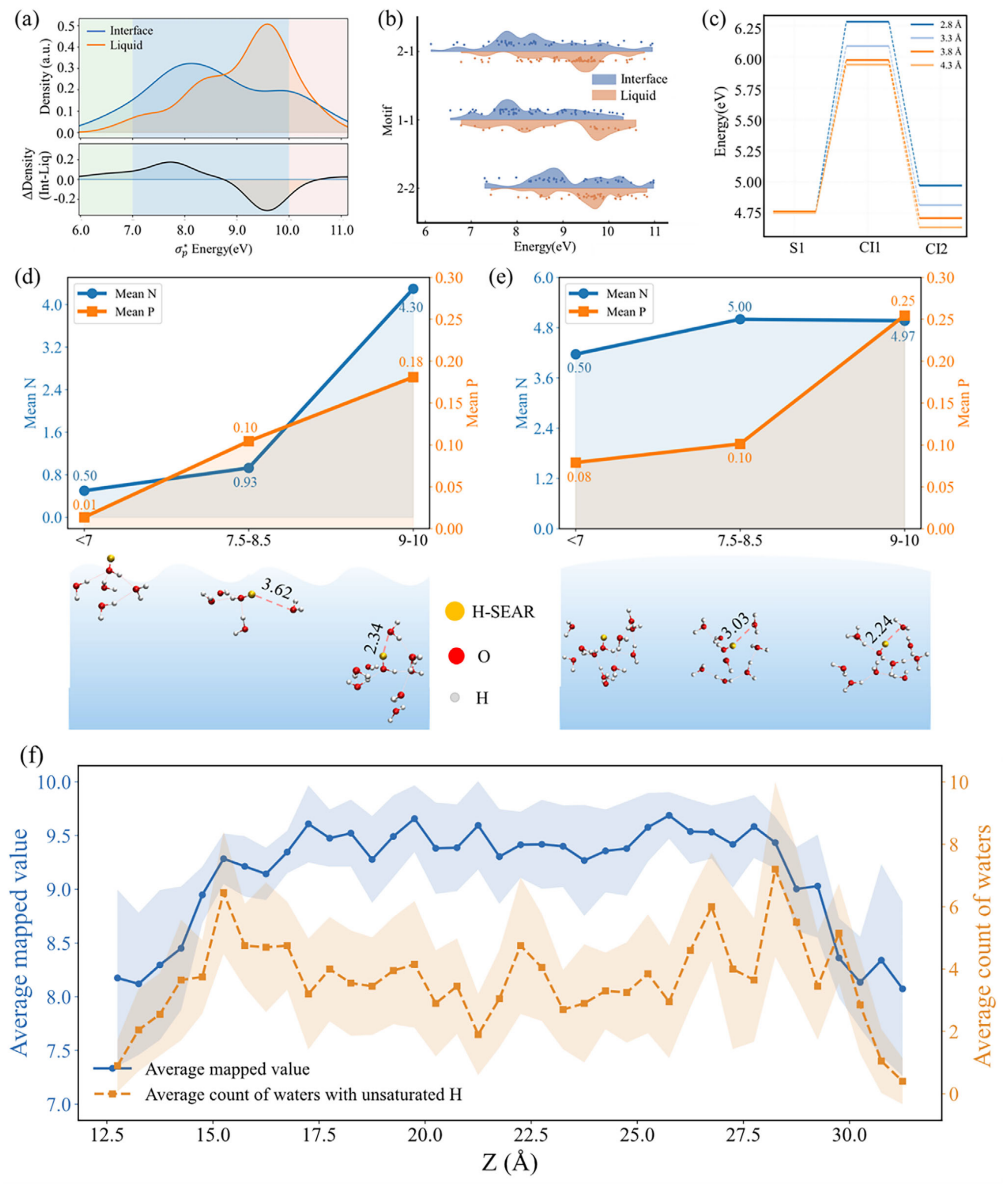
(a) ε($\sigma_{p}^{*}$) distributions for the periodic interfacial and bulk ensembles. (b) Probability distributions of the three most abundant motifs over different ε($\sigma_{p}^{*}$) windows in the two phases. (c) Energies of the excited reactant (S1), the bright/dark conical‐intersection point (CI1), and the dark‐state product (CI2) as functions of the distance to the water molecule hydrogen‐bonded to the phenolic hydroxyl group. Here, H‐SEAR denotes the H atom around which the solvent‐side excess‐electron density is mainly concentrated. (d,e) Variations of N and P across different ε(σ_p_*) windows for the interfacial (d) and bulk (e) phases; representative local structures shown below each ε(σ_p_*) window illustrate the evolution of the hydrogen‐bond network around the electron‐density accumulation site with increasing ε(σ_p_*). Yellow atoms (H‐SEAR) denote the electron‐density accumulation region. (f) Z‐dependent profiles of the mapped H···O pressing metric and the average count of waters containing non‐hydrogen‐bonded H atoms for the interfacial and bulk phases.

Once truncation‐induced bias is removed, a clear phase‐resolved signal emerges. Statistics over 20 mutually separated periodic snapshots show that the interfacial ε(σ_p_*) distribution is both downshifted and broadened relative to bulk water, as displayed in Figure 4a. Interfacial acceptor levels mainly span about 6–9 eV, whereas the bulk distribution is narrower and centered near 9–10 eV. The resulting mean separation is approximately 0.7 eV. Sensitivity tests for the PDOS‐OH–H ranking depth (Figure S21) snapshots number (Figure S23) confirm that the phase‐resolved ε(σ_p_*) separation is robust within physically reasonable parameter ranges. As in the truncated‐cluster analysis, ε(π*) remains nearly unchanged between phases (Figure S9b). The phase dependence of ΔE_FC_ is therefore again controlled primarily by ε(σ_p_*), but here the signal directly reflects the more frequent occurrence of low‐energy σ_p_* acceptor configurations at the interface, implying a lower energetic cost for electron accommodation. Within the present framework, this downshift also biases ΔE_FC_ in the direction of greater accessibility to πσ*‐related dark or crossing regions, although the quantity should still be interpreted as a trend descriptor rather than an absolute rate or barrier.

To explain the origin of the ε(σ_p_*) phase separation, we first examined whether the local hydrogen‐bond‐topology composition of the acceptor sites differs substantially between the two phases. The result shows that the dominant motifs are not fundamentally different between the two phases: both interface and bulk are dominated by the X‐1 family, with the 2‐2 motif as an important secondary component (Figure 4b). The phase separation in ε(σ_p_*) therefore cannot be explained simply by differences in motif composition. The actual difference lies in the energy distributions within the same motif. As shown in Figure 4b, even when the motif identity is fixed, the ε(σ_p_*) windows remain systematically shifted between the two phases: for the two X‐1 motifs, 1‐1 and 2‐1, the interfacial population is concentrated mainly in the 7.5–8.5 eV window, whereas the bulk population is shifted toward 9–10 eV; for the 2‐2 motif, the overall trend is similar, although the phase separation is somewhat weaker. The dominant phase signal thus arises not from motif counting itself, but from systematic shifts of the ε(σ_p_*) energy windows for the same topology under different phase environments. The interface more readily pushes the same motif into lower‐energy regions, whereas bulk water tends to retain it in higher‐energy windows.

We therefore turn to the dominant X‐1 family and partition it into three ε(σ_p_*) windows, <7, 7.5–8.5, and 9–10 eV, to probe the origin of the phase‐dependent distribution of the same topology. For each unoccupied acceptor orbital in a given window, we identify its SEAR and then characterize the surrounding local solvent environment using the descriptors N and P defined in Section S3.3. The resulting interfacial and bulk statistics are shown in Figure 4d,e, respectively.

At the interface, both N and P increase monotonically with ε(σ_p_*), indicating that within the same X‐1 topology, the energetic shift is accompanied by a continuous structure–energy evolution of the SEAR‐centered solvent environment. The low‐energy window is associated mainly with low‐N, low‐P microenvironments, that is, fewer surrounding waters, weaker directional pressing, and more relaxed hydrogen‐bond arrangements, whereas the higher‐energy windows correspond progressively to higher‐N, higher‐P environments, in which the SEAR is more immersed and more strongly constrained. The representative solvent environments shown beneath Figure 4d directly illustrate this trend: as ε(σ_p_*) increases, unsaturated oxygens are found closer to the SEAR‐centered region, and the local environment becomes more crowded and restrictive.

The bulk results show a different pattern (Figure 4e). Across the three windows, N remains generally high and changes only weakly, whereas P also increases with ε(σ_p_*) and is systematically slightly higher than its interfacial counterpart for the same energy window. This indicates that, relative to the number of surrounding waters, the local directional pressing exerted by nearby unsaturated oxygens is more directly associated with ε(σ_p_*). In other words, what determines whether a given topology resides in a lower‐ or higher‐energy window is not simply how many waters surround the SEAR, but whether local defect sites approach the SEAR at shorter distances and with less favorable directional arrangements.

These statistics support a direct structural interpretation. Because the interface differs from bulk water in both microstate supply and hydrogen‐bond‐network relaxability, it more readily provides low‐N, low‐P microenvironments. As a result, the same X‐1 topology is shifted toward lower‐energy acceptor windows at the interface, whereas in bulk water it remains embedded more often in higher‐P, more constrained environments and therefore occupies higher‐energy windows. The phase difference in the energy distributions of the same topology thus originates not from motif identity itself, but from differences in the SEAR‐centered local hydrogen‐bond environment.

To show that this trend is not limited to a selected subset of acceptor orbitals, we further analyzed the last 1000 frames of the interfacial and bulk trajectories along the *z* direction. For each water molecule containing at least one non‐hydrogen‐bonded H, we evaluated the degree of local approach between that H and nearby non‐hydrogen‐bond‐saturated oxygens: an H···O distance of ≤2.0 Å was assigned a value of 1, a distance of ≥4.0 Å a value of 0, and intermediate distances were linearly interpolated and mapped onto the 7–10 range, which was chosen because most of the acceptor‐state energies identified in our previous analyses were concentrated in this window. When both H atoms of the same water were non‐hydrogen‐bonded, the less pressed one was used. The result is shown in Figure 4f. The interfacial non‐hydrogen‐bonded H population is concentrated mainly in the 8–9 range, whereas the bulk counterpart is shifted more strongly toward 9–10, indicating that interfacial defect environments correspond overall to lower‐pressing and lower‐energy conditions for electron accommodation. Meanwhile, the mean occurrence counts of non‐hydrogen‐bonded H are not markedly different between the two phases; given the substantially lower water density at the interface, this instead implies a higher degree of hydrogen‐bond‐network disruption and defect enrichment there. In this sense, the phase separation in ε(σ_p_*) can be understood as the statistical projection of phase differences in the availability and reorganizability of low‐pressing microstates.

The same logic may also provide a complementary perspective on curvature effects in micro‐ and nanodroplet chemistry. In addition to the electric‐field and charge effects often emphasized in such systems, curvature may modify the interfacial hydrogen‐bond network and thus alter the availability and relaxability of defect‐rich, low‐pressure microenvironments. This could, in turn, influence the energetic cost of electron dispersal into the solvent and thereby contribute to variations in reactivity. Our results therefore do not contradict field‐based interpretations, but suggest that curvature‐induced changes in local hydrogen‐bond topology may represent an additional structural factor.

Finally, we use small‐system SA‐CASSCF diagnostics to test whether the above structure–energy relationships remain directionally consistent with crossing‐region behavior (Figure 4c). In phenol–water clusters, the distance between the acceptor‐water oxygen and the phenolic hydroxyl H was progressively shortened to increase local directional pressing, after which O─H elongation scans were carried out to extract the scan‐based CI1 and CI2 indicators. As pressing increases, the S1 vertical excitation energy remains nearly unchanged, consistent with both the cluster and periodic ensemble statistics; by contrast, both CI1 and CI2 rise to varying extents as the local pressing increases, indicating that both the difficulty of reaction initiation and the stability of the product state are directly linked to the local pressing experienced by the dark‐state acceptor orbital. Stronger pressing therefore not only raises the energetic cost of electron accommodation, making product stabilization less favorable, but also increases the energy required to access the dark‐state pathway.

Notably, Table S2 further records the relationship between the position of the CI2 crossing along the O─H elongation coordinate and its corresponding energy. The results show that, as the pressing on the dark‐state acceptor orbital increases, the CI2 crossing appears at progressively more elongated O─H geometries, meaning that a larger O─H extension is required to reach the crossing region. If the pressing continues to increase, the dark‐state surface minimum is expected to rise further relative to S_1_, and the CI2 crossing to shift toward progressively longer O─H bond lengths, potentially falling outside the accessible elongation range. Under such conditions, if the corresponding dark‐state product also becomes less stable than the excited reactant state, the reaction channel would be suppressed and the reaction rate correspondingly affected. This scenario is physically consistent with the relative‐energy trend between CI2 and S1 shown in Figure 4c.

### 4.4 Phase‐Resolved Dual Control and Engineering Implications

At the descriptor level, the periodic‐model statistics support a dual‐control mechanistic picture for πσ*‐mediated photochemistry at aqueous interfaces. For phenol, excitation at 270 nm [14] first populates the ^1^ππ* bright state; under nonadiabatic coupling, the system then evolves into a solvent‐coupled dark state, in which electron density begins to disperse from the phenolic hydroxyl into the surrounding water network. As the O─H bond elongates further, the hydrogen‐bond network around the SEAR reorganizes locally and modulates the energy of the dark‐state acceptor environment. The interfacial–bulk rate difference can thus be viewed as the coupled outcome of two steps: whether the system can access the πσ*‐related dark channel from the FC region, and whether the solvent network can then reorganize favorably enough to stabilize the product‐like dark‐state intermediate relative to the excited reactant. If such stabilization is insufficient, the system is more likely to relax back toward the reactant side; if it becomes sufficiently favorable, O─H bond dissociation can proceed more readily toward products.

**FIGURE 5:**
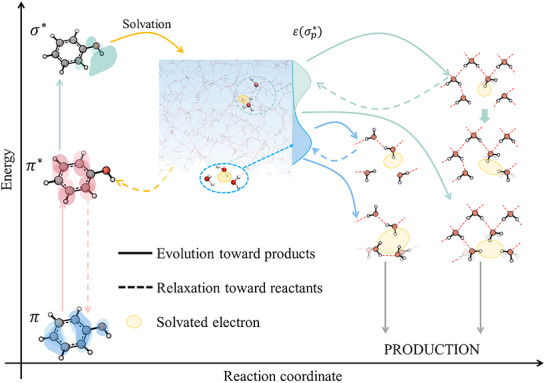
Schematic dual‐control mechanistic picture. Pink and green solid arrows denote photoexcitation and nonadiabatic coupling, respectively. Yellow arrows indicate dark‐state electron transfer from the phenolic hydroxyl to surrounding solvent molecules. Light‐green and blue arrows denote the subsequent solvent‐side reorganization in bulk and at the interface, respectively. Dashed arrows of the same colors indicate the reverse evolution toward the reactant side. The brown–yellow shaded region marks the solvated electron.

Within this picture, the low‐P, low‐ε(σ_p_*) microenvironments identified above acquire a clear mechanistic meaning (as shown in Figure 5). They correspond not only to a lower energetic cost for accommodating transferred electron density, but also to solvent environments in which the hydrogen‐bond reorganization required for stabilization is smaller in extent and therefore easier to achieve. If the local perturbation induced by electron entry does not relax away instantaneously after the electron density recedes, but retains some structural lag, then the same region may become more likely to attract and support dark‐state electron density in subsequent excitation events, progressively biasing the local network toward configurations more favorable for electron stabilization. The interfacial advantage over bulk water can therefore be understood on both counts: the more disrupted and defect‐rich interfacial network enriches lower‐ε(σ_p_*) acceptor environments and lowers the trend barrier for entering the dark‐state pathway, while the lower local coordination and greater relaxability of the interfacial hydrogen‐bond network reduce the extent and cost of reorganization needed for product stabilization. In this sense, the interfacial enhancement arises not only because the reaction is easier to initiate, but also because once initiated, the local solvent environment is more readily driven toward the product side.

From the perspective of multiphase modeling and photochemical engineering, this dual‐control picture provides a useful set of descriptor‐level inputs for phase‐dependent parameterization. The phase‐resolved ε(σ_p_*) distributions and their joint statistics with (N, P) quantify how interfacial microstate supply and hydrogen‐bond‐network reorganizability jointly regulate the formation and persistence of electron‐transfer‐generated products. These quantities can therefore serve as structure‐resolved inputs for incorporating interfacial effects into multiphase reaction descriptions. More broadly, the framework suggests a clear experimental implication: whenever product stabilization depends strongly on electron solvation, the air–water interface is statistically more likely than bulk water to provide lower stabilization cost. Manipulating interfacial defect density and relaxability should therefore offer a practical route for tuning both interfacial reactivity trends and apparent rates. In realistic systems, such tuning variables may include electrolyte concentration, ionic strength, surfactants, local electric fields or applied potentials, and film thickness. Each of these perturbations is expected to influence the abundance and reorganizability of low‐pressing microstates and, in turn, to shift ε(σ_p_*) and ΔE_FC_ systematically.

## 5 Conclusions

We have developed a scalable, structure‐resolved, and statistics‐driven framework for phase‐resolved comparison of πσ*‐related photochemistry at the air–water interface and in bulk water. The framework shows that truncated clusters do not provide a reliable baseline for strict phase comparison, because truncation‐affected boundaries dominate acceptor selection and suppress the intrinsic interface–bulk contrast. In periodic models, this bias is removed, and a clear phase‐resolved signal emerges: the interfacial ε(σ_p_*) distribution is shifted lower in energy by approximately 0.7 eV on average and is substantially broader than its bulk counterpart.

The phase separation is governed primarily not by motif‐count differences, but by systematic within‐motif shifts of ε(σ_p_*) between phases. Low‐N, low‐P microenvironments consistently correlate with lower ε(σ_p_*), identifying weakly constrained, low‐pressing acceptor environments as the structural origin of favorable electron accommodation at the interface.

Together with SA‐CASSCF diagnostics, these results support a phase‐resolved descriptor‐level dual‐control picture in which reactivity depends jointly on initiation accessibility and solvent‐driven product stabilization. The phase‐resolved ε(σ_p_*) distributions and their joint statistics with (N, P) provide descriptor‐level inputs for phase‐dependent multiphase photochemical modeling. More broadly, because ε(σ_p_*) and ΔE_FC_ are governed by local directional constraint and hydrogen‐bond‐network reorganizability, variables that modulate interfacial microstate supply, such as ionic strength, surfactant coverage, applied electric fields, and film thickness, offer practical routes for tuning interfacial photochemical reactivity in engineered multiphase systems.

## Author Contributions

**Jialing Shi**: writing – review and editing, validation, investigation. **Jinping Zhao**: funding acquisition, writing – review and editing. **Qiang Yin**: conceptualization, investigation, methodology, writing – original draft, writing – review and editing, visualization. **Yu Mao**: supervision, writing – review and editing, conceptualization, resources. **Benkun Tan**: writing – review and editing. **Ziyun Wang**: resources, writing – review and editing. **Chengjun Li**: funding acquisition, writing – review and editing. **Da Wang**: supervision, writing – review and editing, funding acquisition. **Yongbo Xie**: writing – review and editing.

## Conflicts of Interest

The authors declare no conflict of interest.

## Supporting information




**Supporting File**: advs76249‐sup‐0001‐SuppMat.docx.

## Data Availability

The data that supports the findings of this study are available in the supplementary material of this article.
