# Supplementary material for: Phase‐Resolved Dual Control of Phenol Photodissociation at the Air–Water Interface From Structure‐Resolved Statistics
Source: Adv Sci (Weinh). 2026 Jun 22:e76249. Online ahead of print. doi: 10.1002/advs.76249 (PMC13336552; doi:10.1002/advs.76249)
Supplement: Supplementary file 1 — Supporting File: advs76249‐sup‐0001‐SuppMat.docx. [file ADVS-9999-e76249-s001.docx]

**Supporting Information**

**Phase-resolved dual control of phenol photodissociation at the air–water interface from structure-resolved statistics**

Qiang Yin^1#^ , Jialing Shi^1#^, Jinping Zhao^2^, Chengjun Li^2^, Yongbo Xie^3^, Benkun Tan^4^, Da Wang^1^*, Ziyun Wang^5^, Yu Mao^5^*

1 School of Materials and Energy, Central South University of Forestry and Technology, Changsha, 410004, China

2 College of Landscape Architecture, Central South University of Forestry and Technology, Changsha, 410004, China

3 Testing Technology Company of Changsha Research Institute of Mining and Metallurgy Co., Ltd., Changsha 410000, China

4 School of Civil Engineering and Architecture, Hunan University of Arts and Science, Changde 415000, China

5 School of Chemical Sciences, University of Auckland, Auckland 1010, New Zealand.

*Corresponding authors:

[yxwang2006@yeah.net](mailto:yxwang2006@yeah.net) (D Wang); [yu.mao@auckland.ac.nz](mailto:yu.mao@auckland.ac.nz) (Y Mao)

#Qiang Yin and Jialing Shi contributed equally to this work.

**Contents**

[S1. Classical MD simulations and snapshot sampling 3](#_Toc229673975)

[S2. AIMD setup, structural validation, and snapshot selection 3](#_Toc229673976)

[S3. Cluster extraction protocol and region definition 5](#_Toc229673977)

[S4. Electronic-structure details 8](#_Toc229673978)

[S4.1 Electronic-structure calculations for cluster models (Gaussian) 8](#_Toc229673979)

[S4.2 Electronic-structure calculations for periodic models (CP2K) 8](#_Toc229673980)

[S5. SA-CASSCF details 9](#_Toc229673981)

[S5.1 Validation of the SA-CASSCF description for gas-phase phenol 9](#_Toc229673982)

[S5.2 Active-space selection and state tracking 10](#_Toc229673983)

[S6. Validation: orbital-gap proxy for excitation energies 12](#_Toc229673984)

[S7. Definition of the dark-state acceptor sampling window: PDOS criteria, contamination control, and upper-energy termination 13](#_Toc229673985)

[S7.1. PDOS definitions and practical implementation 13](#_Toc229673986)

[S7.2. Sources of contamination and fragment-projected removal criteria 15](#_Toc229673987)

[S7.3. High-energy water $\boldsymbol{\sigma}*$signals and definition of the upper sampling limit 16](#_Toc229673988)

[S7.4. Final workflow for selecting $\boldsymbol{\sigma p}*$candidates 19](#_Toc229673989)

[S7.5. Representative screening examples in minimal models 21](#_Toc229673990)

[S8. Density-response probe $\boldsymbol{\Delta}\boldsymbol{\rho}$: definition, orbital matching, and SEAR localization 23](#_Toc229673991)

[S8.1. Rationale for introducing $\boldsymbol{\Delta}\boldsymbol{\rho}$ 23](#_Toc229673992)

[S8.2. Construction of $\boldsymbol{\Delta}\boldsymbol{\rho}$and the choice of $\boldsymbol{\Delta}\boldsymbol{r}$ 23](#_Toc229673993)

[S8.3. Orbital matching and phase handling 24](#_Toc229673994)

[S8.4. SEAR definition and acceptor-site localization 25](#_Toc229673995)

[S8.5. Representative $\boldsymbol{\Delta}\boldsymbol{\rho}$maps in minimal and cluster models 27](#_Toc229673996)

[S9. Motif classification and local descriptors 30](#_Toc229673997)

[S10. Robustness and sensitivity analysis of operational definitions 32](#_Toc229673998)

[S10.1. Rationale and validation of the upper sampling cutoff E_cut_ 32](#_Toc229673999)

[S10.2. Sensitivity to the PDOS-OH–H ranking depth k 34](#_Toc229674000)

[S10.3. Rationale and sensitivity of the SEAR-centered geometry used for P 37](#_Toc229674001)

[S10.4. Statistical robustness and representativeness of the periodic snapshots 38](#_Toc229674002)

[References 42](#_Toc229674003)

# S1. Classical MD simulations and snapshot sampling

Classical molecular dynamics (MD) simulations were carried out in GROMACS^[1]^ (2023.5) to generate representative bulk aqueous and air–water interfacial environments of phenol. Bulk water was simulated in a cubic box of ~40 Å. Interfacial systems were constructed by adding ~30 Å vacuum along the surface normal on top of an equilibrated bulk water slab, and then propagated under NVT conditions to mimic the air–water interface.

Phenol was described by GAFF, while water was modeled with the OPC (4-site) water model. Phenol atomic charges were derived using a RESP2^[2]^ protocol: (i) geometry optimization in the liquid phase followed by RESP fitting, (ii) single-point calculation in the gas phase followed by RESP fitting, and (iii) averaging the two RESP charge sets. All charge calculations were performed with Gaussian 16 at the B3LYP/def2-TZVP level.

The temperature was maintained at 298.15 K using the velocity-rescale (v-rescale) thermostat^[3]^. For the bulk phase, the pressure was coupled to 1 bar using the Parrinello–Rahman barostat^[4]^. A time step of 1 fs was used, and configurations were saved every 1 ps to compressed trajectories (xtc). Van der Waals and real-space electrostatics used a cutoff of 1.5 nm, while long-range electrostatics were treated with PME. Dispersion corrections were applied to energy and pressure (DispCorr = EnerPres). Water geometry was constrained with SETTLE, and phenol bonds were constrained using LINCS (constraints = hbonds).

Each system was equilibrated for 5 ns. Production sampling lasted 20 ns, run as NPT for bulk water and NVT for the interfacial slab. In total, ~20,000 frames were collected for analysis. For cluster extraction, 30 snapshots were selected at equal time intervals from each phase.

# S2. AIMD setup, structural validation, and snapshot selection

Additional ab initio MD (AIMD) simulations were performed using CP2K (2023.1) to refine solvent configurations for periodic electronic-structure analysis. AIMD employed the PBE exchange–correlation functional with DFT-D3(BJ) dispersion correction, using DZVP-MOLOPT basis sets with GTH pseudopotentials.

To check whether the AIMD trajectories retained physically reasonable liquid and interfacial water structures, structural analyses were performed on the equilibrated portions of the trajectories. Figure S1a illustrates the definition of the orientational parameter^[5]^ used in Figures S1c,d. For each O–H bond, the orientation relative to the laboratory $z$axis was described by

$$\cos\theta_{OH,z}=\frac{\vec{r}_{\mathrm{OH}}\cdot\hat{z}}{\mid\vec{r}_{\mathrm{OH}}\mid} (1)$$

where positive and negative values correspond to O–H bonds pointing along $+z$and $-z$, respectively.

The O–O radial distribution functions are shown in Figure S1b. Both the bulk and interfacial water models show a physically reasonable nearest-neighbor O···O distance, with the first peak located in the normal liquid-water range. Compared with bulk water, the interfacial layer exhibits a lower and broader first-shell feature, consistent with a more heterogeneous and partially disrupted hydrogen-bond environment at the air–water boundary.

The $z$-resolved O–H orientational profiles further distinguish the two phases. In the bulk model (Figure S1c), $\left\langle\cos\theta_{OH,z} \right\rangle$fluctuates around zero without a persistent spatially localized orientation, consistent with orientational cancellation in an approximately isotropic liquid environment. In the interfacial model (Figure S1d), nonzero orientational features appear near the two water–vacuum boundaries, with opposite signs at the two sides of the slab. This behavior is expected for a double-interface system because the outward surface normals point in opposite directions at the upper and lower interfaces. The middle region of the slab remains close to zero, indicating that the net orientation is localized mainly at the interfacial layers.

These RDF and orientational analyses indicate that the AIMD trajectories reproduce the essential structural characteristics of the corresponding phases: locally reasonable water–water coordination in both systems, near-isotropic orientational cancellation in the bulk phase, and surface-normal orientational anisotropy at the interface. From the last 3000 frames of each AIMD trajectory, 20 statistically separated snapshots were selected for periodic single-point calculations and subsequent PDOS/$\Delta\rho$ analysis.


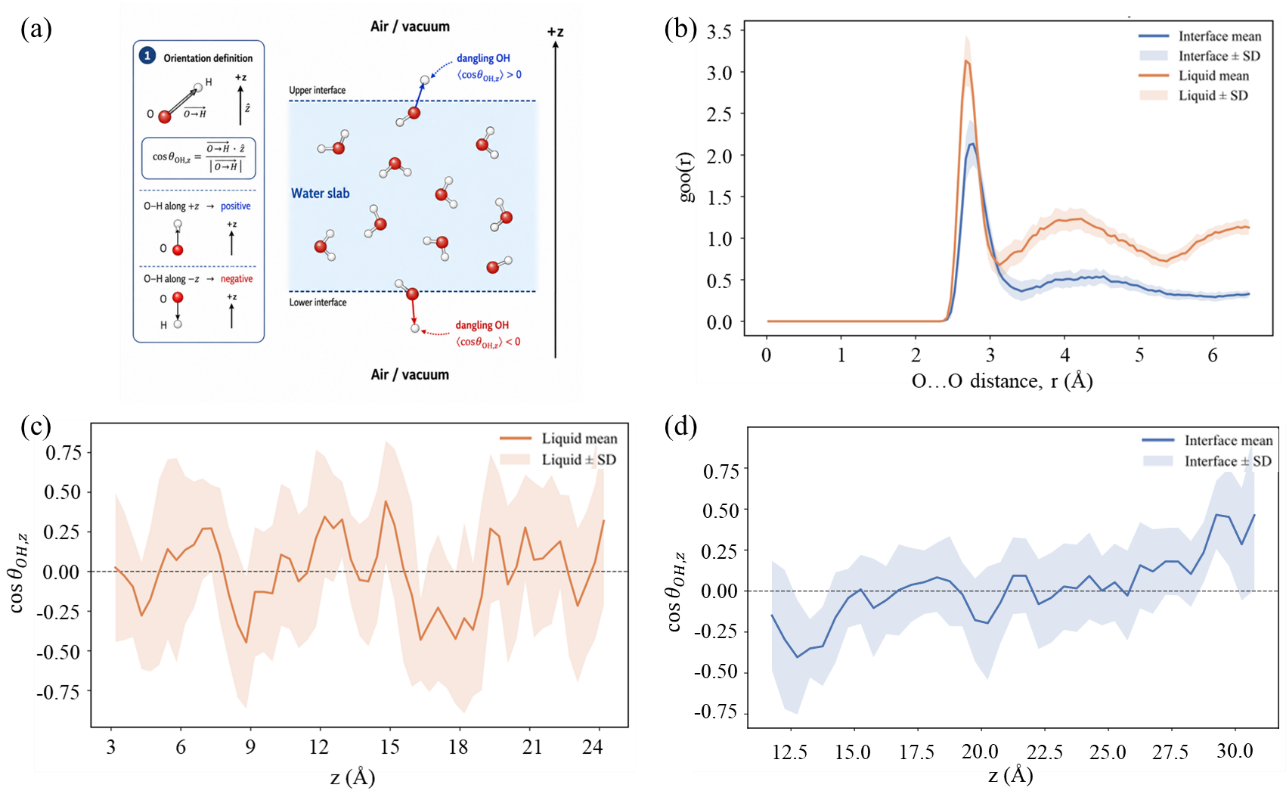


Figure S1 (a) Definition of the O–H orientational parameter $\cos\theta_{\mathrm{OH},z}$. (b) O–O radial distribution functions of bulk and interfacial water. (c,d) $z$-resolved O–H orientational profiles for bulk and interfacial models, respectively.

# S3. Cluster extraction protocol and region definition

Cluster models were carved from MD snapshots using a simple center-of-mass (COM) criterion. For each snapshot, a water molecule was retained (with its full geometry) if the distance between its oxygen atom and the phenol center of mass satisfied r ≤ 10 Å; otherwise it was discarded. The 10 Å cutoff was guided by radial distribution functions (RDFs): Figure S2 compiles RDFs of water oxygens around selected phenolic sites (ring C atoms and the hydroxyl O/H). In all cases, the RDFs become essentially flat beyond ~8 Å, indicating diminished structural variation at larger distances. Because the extraction radius is defined with respect to the phenol COM (rather than a local atom), a conservative buffer was adopted to avoid undercutting the solvation environment on the hydroxyl side, leading to the uniform choice of 10 Å. The RDF comparison further supports the interfacial phenol geometry (Figure S3a): sites proximal to the hydroxyl side (C2/C3/C4/O12/H13) show smaller interface–bulk differences than distal sites (C1/C5/C6), consistent with a hemi-immersed, OH-down orientation of phenol at the air–water interface^[6]^. In addition, an interfacial thickness estimated from the density profile using the 0.1–0.9 ρ_bulk_ criterion yields ~3.8 Å (Figure S3b), consistent with typical literature values (~3.5-4 Å scale) ^[7]^, supporting the validity of the MD setups.

To define interior vs boundary regions in truncated clusters, we introduced a normalized phenol-centered distance ξ = r / r_max_, where r is the phenol-COM to water-oxygen distance. Because truncated clusters are not perfectly spherical (because, during cluster extraction, we deliberately retained more water molecules in the vicinity of the phenolic hydroxyl group), r_max_ cannot be represented by a single constant. We therefore used a ray-based envelope normalization: rays were uniformly emitted from the phenol COM; each oxygen atom was projected onto its nearest ray direction, and the maximum projected distance along that direction was taken as r_max_ for that ray. Bulk clusters used 30 rays (full-space coverage), while interfacial clusters used 15 rays (hemispherical coverage). The resulting ξ definition is stable with respect to the selected-atom spatial distribution. In subsequent statistics, ξ > 0.8 is used to define a boundary-like region, whereas ξ ≤ 0.8 defines an interior (bulk-like) region.


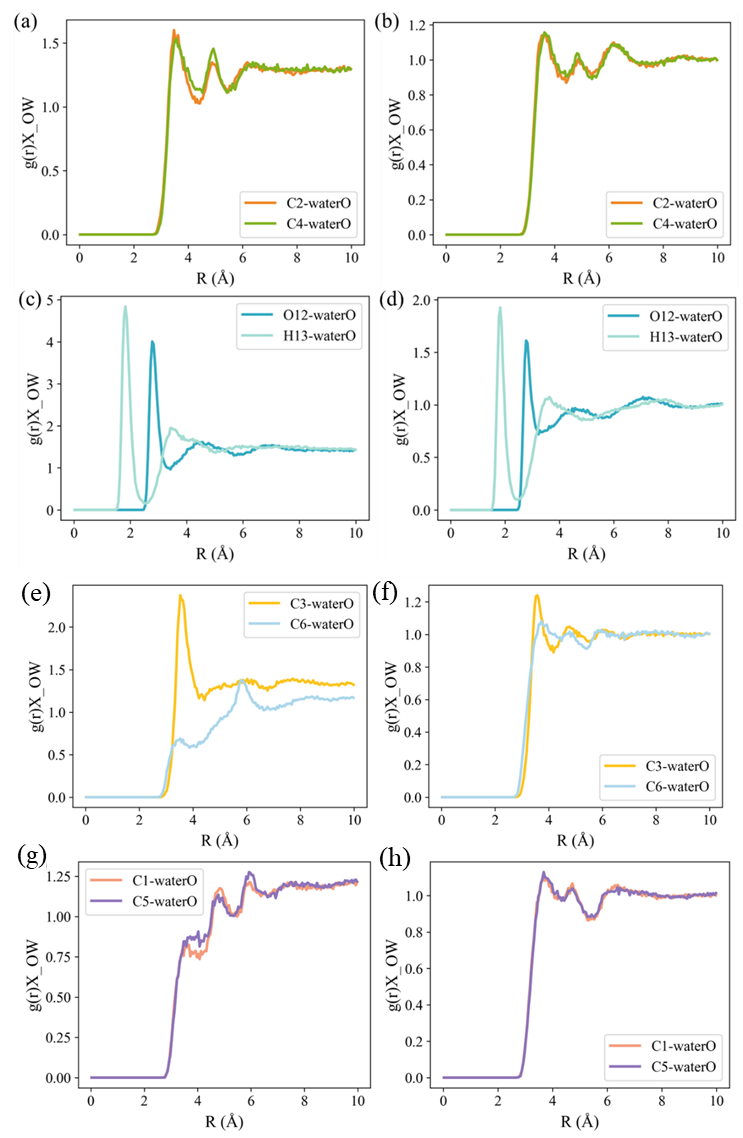


Figure S2. Radial distribution functions (RDFs) of water oxygens around phenol. Panels (a,c,e,g) correspond to the air–water interfacial clusters, whereas (b,d,f,h) correspond to the bulk-aqueous clusters. The specific phenol atom pairs associated with each RDF are indicated in the legends of the respective panels.


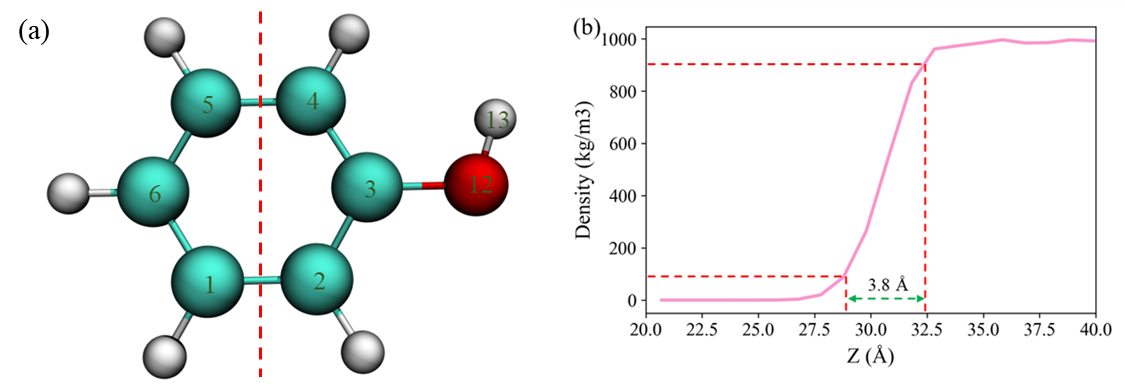


Figure S3. Site partitioning of phenol and determination of the interfacial thickness. (a) Phenol structure schematic highlighting two sets of sites: the “OH-distal” side (C5, C6, C1; left of the red divider) and the “OH-proximal” side (C4, C3, C2, O12, H13; right of the divider), used to rationalize site-dependent solvation signatures. (b) Magnified local density profile across the air–water boundary, used to identify the interfacial region and to estimate the interfacial thickness (defined by the 0.1ρ_bulk_–0.9ρ_bulk_ criterion).

# S4. Electronic-structure details

## S4.1 Electronic-structure calculations for cluster models (Gaussian)

Ground-state single-point calculations and vertical excitation energies for all truncated cluster models were computed with Gaussian 16 using B3LYP/def2-TZVP. Excited-state energies were obtained within TDDFT by requesting 50 singlet excited states (NStates = 50) to ensure that the target excitation characters were included in the computed manifold.

## S4.2 Electronic-structure calculations for periodic models (CP2K)

Periodic single-point electronic-structure calculations were performed with CP2K under 3D periodic boundary conditions. To obtain a stable and consistent starting point for subsequent hybrid calculations, each configuration was first converged at the PBE level using the TZV2P-MOLOPT-GTH-q4 basis and the corresponding GTH pseudopotentials, and the resulting wavefunction (WFN restart) was then reused as the initial guess for hybrid calculations.

Hybrid calculations were carried out at the PBE0 level using the auxiliary density matrix method (ADMM) with TZV2P-MOLOPT-GTH basis sets, corresponding GTH-PBE pseudopotentials, and element-specific ADMM auxiliary fitting basis sets. ^[8]^ The hybrid setup used a 25% exact-exchange fraction and Grimme-type dispersion correction (DFT-D3(BJ), referenced to PBE0). The real-space grid employed a multigrid scheme (NGRIDS = 5) with a plane-wave cutoff of CUTOFF = 450 Ry and REL_CUTOFF = 50 Ry. Self-consistent-field convergence used EPS_SCF = 5×10⁻⁶ and up to MAX_SCF = 128 iterations, with RESTART wavefunctions as the initial guess.

To reduce the computational cost of exact exchange, a truncated Coulomb operator was used with a cutoff radius of 6.0 Å, together with Schwarz screening (EPS_SCHWARZ = 1×10⁻⁶). To ensure sufficient virtual-orbital coverage for the PDOS analysis, ADDED_MOS = 500 virtual MOs were included. Where needed for orbital inspection, Molden-format orbitals were exported via MO_MOLDEN.

# S5. SA-CASSCF details

## S5.1 Validation of the SA-CASSCF description for gas-phase phenol

To assess whether the SA-CASSCF protocol used in the main text provides a chemically reasonable description of the phenol excited states, we first benchmarked the gas-phase phenol reference against available experimental and theoretical data. At the S_0_ equilibrium geometry, SA-CASSCF(8,8)/6-31+G(d,p) gives vertical excitation energies of 4.73 and 5.76 eV for S_1_($\pi\pi^{*}$)and S_2_($\pi\sigma^{*}$), respectively. These values are in reasonable agreement with the experimental reference values of 4.51 and 5.12 eV and with previous calculations, which typically place S_1_ in the 4.5–5.0 eV range and S_2_ in the 5.4–5.7 eV range^[9]^. Along the O–H elongation coordinate, the S_2_/S_1_ crossing (denoted CI1 in the present work) and the S_2_/S_0_ crossing (CI2) occur at approximately 1.2 and 1.6 Å, respectively, in good agreement with previous reports for phenol^[10]^. The CI1 barrier obtained at this level is somewhat higher than values reported from calculations using larger basis sets and/or higher-level treatments, but remains consistent with results obtained at comparable SA-CASSCF basis-set levels^[9]^. Because the purpose of the present CASSCF calculations is to validate trend direction rather than to establish a quantitatively converged crossing barrier, this level of agreement is sufficient for the mechanistic support sought here.

## S5.2 Active-space selection and state tracking

The chosen (8,8) active space contains the six phenyl π/π* orbitals together with the O–H σ and σ* orbitals, which are the essential orbitals required to describe the ππ* and πσ* states relevant to the present analysis. Representative active orbitals are shown in Figure S4. The dominant configuration-interaction coefficients of the state-averaged CASSCF wavefunctions at the key geometries are summarized in Table S1. Across the S_0_ minimum, CI1, and CI2 geometries, the identities of the target excited states remain qualitatively unchanged. S_1_ is consistently dominated by ππ* configurations, mainly involving MO4→MO7 (~47%) and MO3→MO7 (~32%) excitations, whereas S_2_ remains dominated by the MO4→MO5 excitation (~86%) and is therefore assigned as the πσ* state. These results show that the selected active space preserves the physically relevant orbital manifold and that no qualitative state switching occurs for the target states along the geometries relevant to the present analysis.


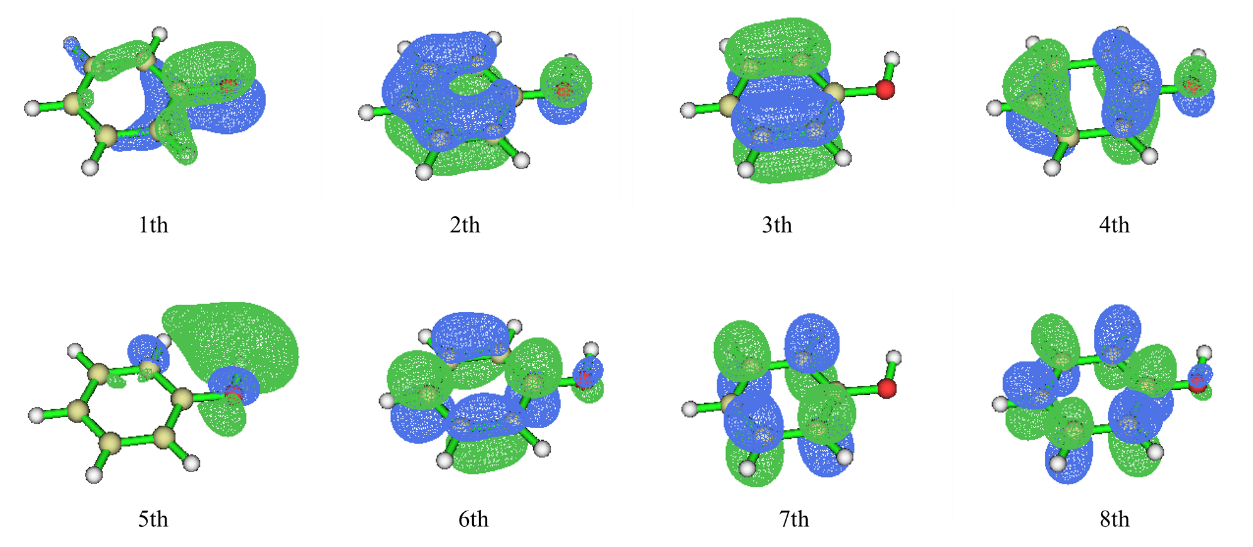


Figure S4: Illustration of the 1st to 8th active orbitals

Table S1: Principal configuration interaction coefficients of the CASSCF wavefunctions for the S_1_ and S_2_ states of the isolated phenol at S1, CI1 and CI2 geometries. See Figure S4 for the characters of active MOs.

|  | S1 | | CI1 | | CI2 | |
| --- | --- | --- | --- | --- | --- | --- |
| Configuration | S1 | S2 | S1 | S2 | S1 | S2 |
| 111ab000 | **0.933** | 0.000 | **0.861** | 0.000 | **0.901** | 0.000 |
| 111a00b0 | 0.000 | **0.687** | 0.000 | **0.678** | 0.000 | **0.728** |
| 11a100b0 | 0.000 | **0.565** | 0.000 | **-0.559** | 0.000 | **0.455** |

Table S2 Evolution of the positions and energies of the CI1 and CI2 crossing points as the water molecule near the phenolic hydroxyl group approaches the O–H site.

|  | CI1 | | CI2 | |
| --- | --- | --- | --- | --- |
| Distance | Len(Å) | Energy(eV) | Len(Å) | Energy(eV) |
| 2.8 | 1.253 | 6.23 | 1.632 | 4.97 |
| 3.3 | 1.221 | 6.09 | 1.595 | 4.81 |
| 3.8 | 1.204 | 5.98 | 1.561 | 4.71 |
| 4.3 | 1.202 | 5.94 | 1.513 | 4.63 |

To test whether the bright–dark vertical-excitation energy separation can be used to track the CI1-related barrier, we performed additional short scans (Figure S5a) by further shifting the nearby water molecule relative to the geometries in Table S2, locating only the CI1 point in each case. For the two additional paths, the distance from the nearby water molecule to the phenolic hydroxyl H was varied from 4.0 to 3.0 Å in 0.5 Å increments. The resulting S_2_–S_1_ energy differences and CI1 energies show a clear linear correlation (Figure S5b), with a fitted $R^{2}=0.953$. This result supports the use of the bright–dark excitation-energy separation as a trend-level indicator of the CI1 barrier.


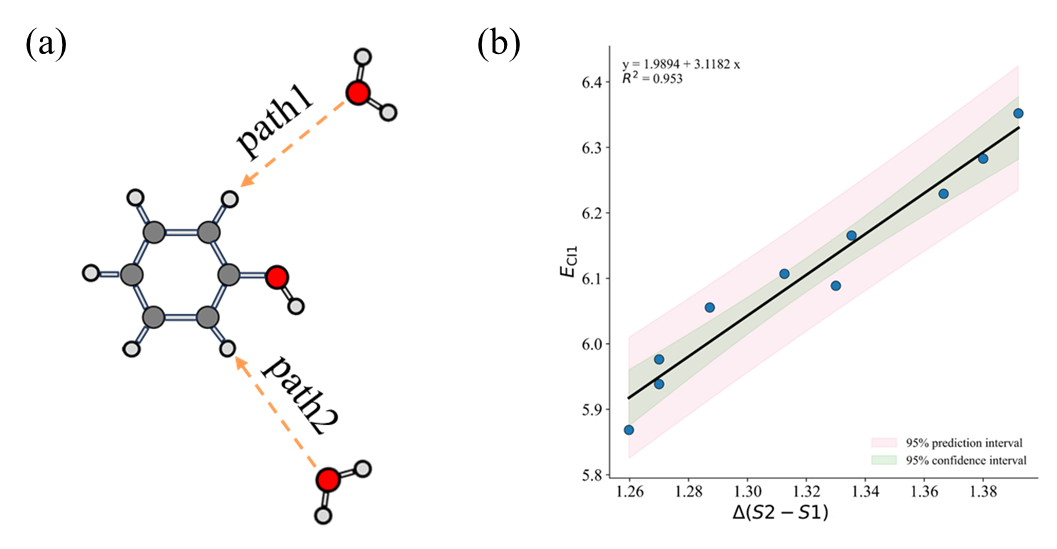


Figure S5. (a) Two additional scan paths used to vary the position of the nearby water molecule relative to the phenolic hydroxyl H. (b) Correlation between the S_2_–S_1_ vertical-excitation energy difference and the CI1 energy, supporting the use of the bright–dark energy separation as a trend-level proxy for the CI-related barrier.

# S6. Validation: orbital-gap proxy for excitation energies

We validated the use of ground-state donor–acceptor orbital gaps as a proxy for vertical excitation energies on MD-extracted phenol–water clusters (30 interfacial and 30 bulk snapshots). TDDFT calculations were carried out with Gaussian 16 at the B3LYP/def2-TZVP level (50 singlet states). For each selected excitation, the corresponding orbital energy gap Δε was computed from the same single-point calculation, using the donor–acceptor orbital pair that dominates the TDDFT transition. To extend the validation range, five additional structures were randomly selected from each phase, and the number of singlet states was increased from 50 to 200.

The π→π* channel was assigned by the phenol-centered HOMO(π)/LUMO(π*) pair and the TDDFT state dominated by this excitation (typically also exhibiting comparatively large oscillator strength). For the π→σ* channel, one representative state was selected from each of the low/mid/high σ* energy windows, requiring a dominant single π→σ* configuration with >85% weight (squared TDDFT coefficient) to ensure mode purity.

As shown in Figure S6, TDDFT excitation energies (x-axis) correlate linearly with Δε (y-axis) for both π→π* and π→σ* excitations in both phases (R² > 0.95), supporting the orbital-gap proxy when excitation character is held fixed.


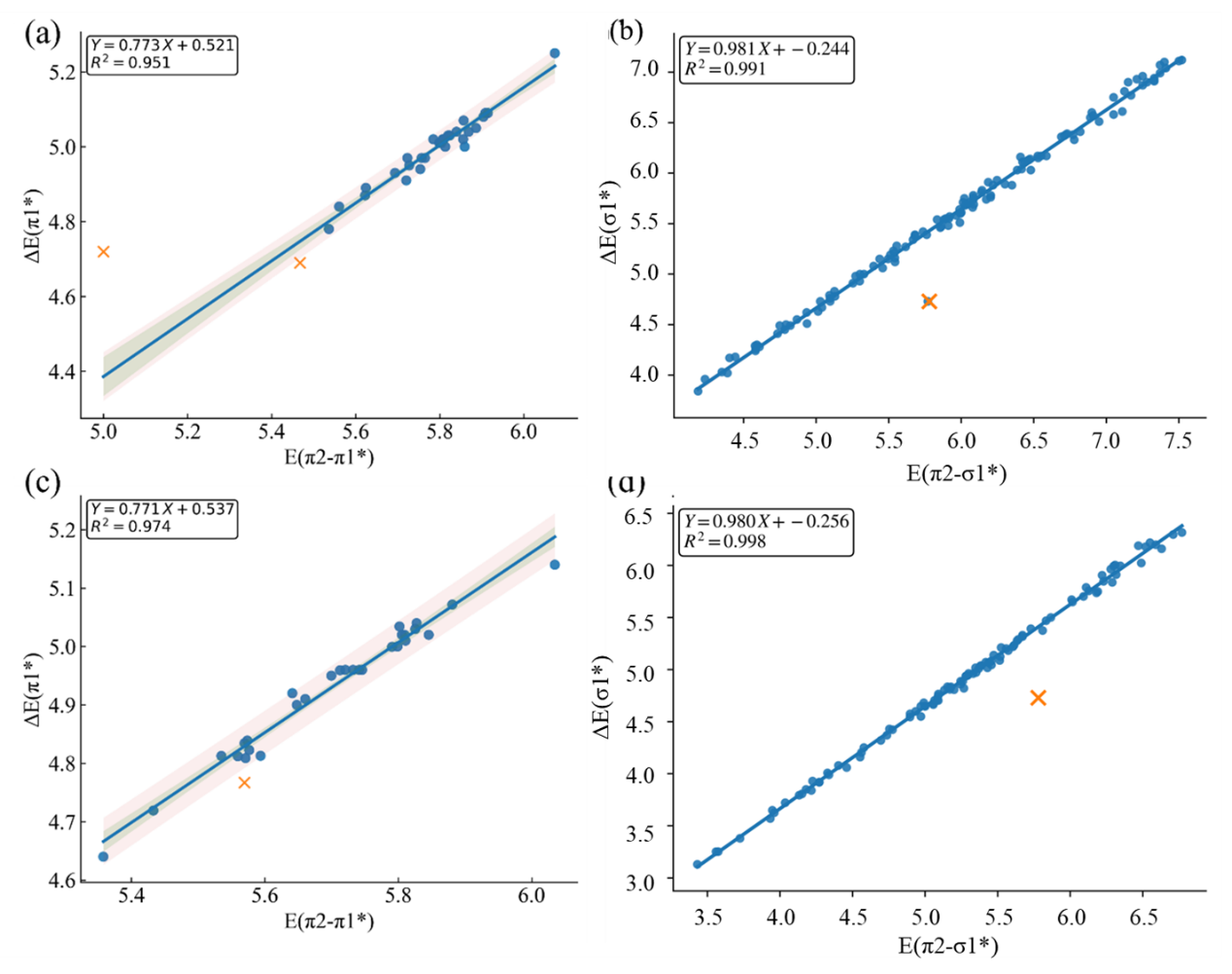


Figure S6. Orbital-gap proxy for excitation energies. Linear correlation between TDDFT vertical excitation energies (x-axis, eV) and donor–acceptor orbital energy gaps Δε (y-axis, eV) for (a,b) interfacial clusters and (c,d) bulk clusters (30 snapshots each). (a,c) π→π* excitations assigned by the HOMO(π)/LUMO(π*) pair; (b,d) π→σ* excitations selected from low/mid/high σ* windows with >85% single-configuration weight. Solid lines: linear fits; shaded regions: 95% confidence interval (green) and 95% prediction interval (pink).

# S7. Definition of the dark-state acceptor sampling window: PDOS criteria, contamination control, and upper-energy termination

## S7.1. PDOS definitions and practical implementation

To enable reproducible and transferable identification of the solvent-stabilized dark-state acceptor manifold $\left\{ \sigma_{p}^{*} \right\}$, we use density-of-states (DOS) and partial density-of-states (PDOS) analyses to convert orbital-shape inspection into numerical screening criteria.

For an isolated system, the total density of states (TDOS) can be written as

$$\mathrm{TDOS}(E)=\sum_{i} \delta(E-\varepsilon_{i}) (2)$$

where $\varepsilon_{i}$is an eigenvalue of the single-particle Hamiltonian and $\delta$is the Dirac delta function. Replacing $\delta$with a broadening function $F(x)$(e.g., Gaussian, Lorentzian, or pseudo-Voigt) yields a broadened TDOS curve.

In the present work, a normalized Gaussian broadening function is used:

$$G\left( x \right)=\frac{1}{c\sqrt{2\pi}}\exp\left( - \frac{x^{2}}{2c^{2}} \right) (3)$$

with

$$c=\frac{\mathrm{FWHM}}{2\sqrt{2\ln2}} (4)$$

where FWHM is the full width at half maximum, an adjustable parameter in Multiwfn. Larger FWHM values produce smoother TDOS curves but at the cost of losing more discrete-level information. Since the present analysis does not focus on the TDOS curve itself, but instead on the energy and composition of individual unoccupied orbitals, a small FWHM value (0.01 eV) was adopted in all DOS-related calculations in order to preserve as much discrete-level detail as possible.

Compared with TDOS, fragment-projected PDOS is more suitable for resolving how specific orbital components are distributed across different energy windows. For a fragment $A$, the PDOS is defined as

$$\mathrm{PDOS}_{A}\left( E \right)=\sum_{i} \Theta_{i,A}F\left( E-\varepsilon_{i} \right) (5)$$

where $\Theta_{i,A}$denotes the fractional contribution of fragment $A$to orbital $i$. Note that the term “projected DOS” used in part of the literature is, in the present context, essentially equivalent to partial DOS.

Ideally, if fragment $A$could be defined directly as the phenolic $\sigma(\mathrm{OH})^{*}$orbital, one could quantify the contribution of phenolic $\sigma(\mathrm{OH})^{*}$character in every unoccupied orbital and thereby determine into which energy window the phenolic $\sigma(\mathrm{OH})^{*}$-related electron density is preferentially transferred. In the current version of Multiwfn, however, PDOS fragments cannot be defined directly in terms of chemical bonds. Because the electron density of the phenolic $\sigma(\mathrm{OH})^{*}$orbital is concentrated mainly on the hydroxyl H atom (Figure S7b,e), we therefore use the hydroxyl H atom as fragment $A$and define the resulting quantity as PDOS-OH–H, which serves as a practical surrogate for phenolic $\sigma(\mathrm{OH})^{*}$character.


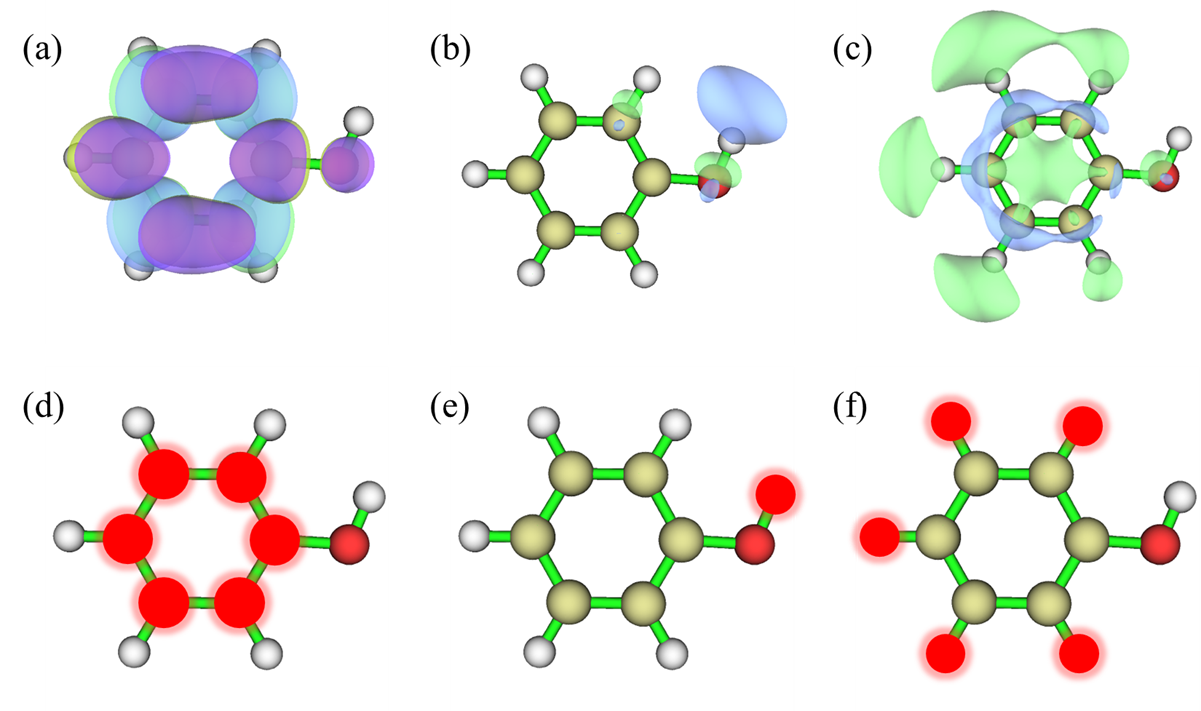


Figure S7. Definition of three fragment-projection PDOS criteria. (a–c) Representative isosurfaces of π*, σ(OH)*, and σ(C–H)* orbitals (π1*/π2* shown in different colors). (d–f) Corresponding selected-atom sets highlighted in red for ring-C, OH–H, and ring-H, respectively.

## S7.2. Sources of contamination and fragment-projected removal criteria

In realistic solvation environments, PDOS-OH–H alone is not sufficient to uniquely identify $\left\{ \sigma_{p}^{*} \right\}$, because two major classes of phenol-derived false positives are present: aromatic $\pi^{*}$orbitals and $\sigma^{*}(\mathrm{CH})$orbitals.

These orbitals can exhibit non-negligible, and occasionally large, PDOS-OH–H values because their virtual-orbital composition often contains mixed contributions from phenolic O–H antibonding character. At the same time, these orbitals are strongly localized in real space. In larger solvation environments, such mixed contributions can accumulate to a level that is no longer negligible, so that a naive PDOS-OH–H-only ranking would inevitably mix target $\sigma_{p}^{*}$candidates with non-target orbitals.

From the real-space orbital-density distributions, the two classes of interference show relatively clear morphological features:

- $\pi^{*}$interferences are primarily characterized by antibonding density distributed over the aromatic carbon framework (Figure 76a,d);
- $\sigma^{*}(C-H)$interferences are primarily characterized by antibonding density along the aromatic C–H bond directions (Figure S7c,f).

We therefore introduce two additional fragment-projection criteria, defined in the same spirit as PDOS-OH–H:

- all aromatic ring C atoms are grouped to define PDOS-ring–C, used to identify and reject $\pi^{*}$interferences;
- all aromatic ring H atoms are grouped to define PDOS-ring–H, used to identify and reject $\sigma^{*}(C-H)$interferences.

Accordingly, identification of the target dark-state acceptor orbitals no longer relies on PDOS-OH–H alone, but instead on three complementary criteria: PDOS-OH–H is used to collect candidate orbitals, whereas PDOS-ring–C and PDOS-ring–H are used to reject $\pi^{*}$and $\sigma^{*}(C-H)$false positives, respectively.

## S7.3. High-energy water $\boldsymbol{\sigma}^{\boldsymbol{*}}$signals and definition of the upper sampling limit

In addition to the phenol-derived $\pi^{*}$and $\sigma^{*}(C-H)$interferences, solvent water molecules themselves introduce another important class of high-energy contamination, namely high-energy water $\sigma^{*}$-like orbitals. Based on inspection of minimal cluster models, water molecules exhibit not only the lower-energy O–H antibonding orbitals relevant to the present analysis (the “first-$\sigma^{*}$” family), but also another higher-energy class of $\sigma^{*}$-like orbitals whose oxygen-end density pattern is qualitatively different. We refer to this higher-energy class as the second**-**$\sigma^{*}$ water signal. Figure S8 contrasts representative isosurfaces of the first- and second-$\sigma^{*}$ water orbitals and provides an operational morphological reference for distinguishing them, thereby helping to avoid inadvertent inclusion of high-energy dense manifolds in the $\sigma_{p}^{*}$statistics.

From a physical standpoint, we do not exclude the possibility that these high-energy second-$\sigma^{*}$ water orbitals could also accept electron density released from the phenol dark state and thus participate in another type of CI-related crossing behavior. However, because the electronic structure in the high-energy region is considerably denser and more complex, the present work focuses first on establishing the statistical behavior of the lower-energy dark-state acceptor manifold as clearly as possible. Within this $\sigma_{p}^{*}$framework, the high-energy water $\sigma^{*}$signals are therefore treated as an additional source of contamination that should be screened out.


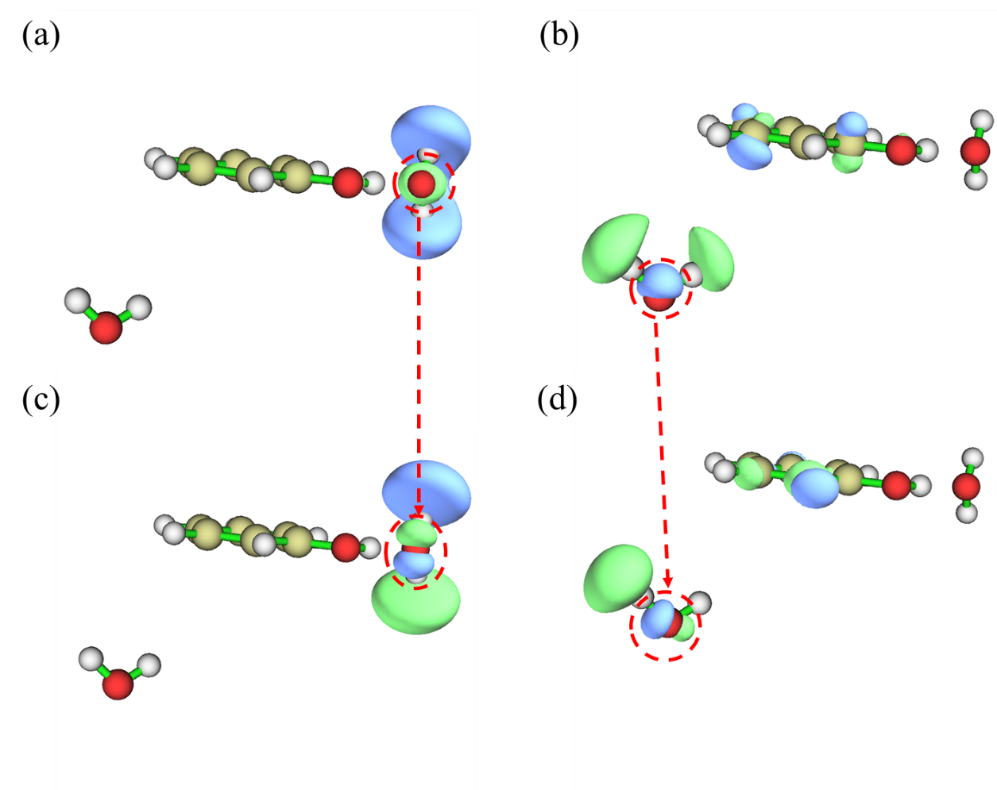


Figure S8. Morphological contrast between first-σ* and second-σ* water acceptor orbitals. (a,b) First-σ*; (c,d) second-σ*. Red circles highlight the most diagnostic differences near the oxygen end.

This type of contamination cannot be removed through additional fragment definitions alone, so we introduce an upper energy limit for the dark-state acceptor sampling window. Inspection of minimal cluster models shows that frequent appearance of the high-energy second-$\sigma^{*}$ water signals typically begins only after the $\sigma^{*}(C-H)$feature becomes pronounced. We therefore use the $\sigma^{*}(C-H)$region to define the upper bound of the dark-state acceptor sampling window, thereby avoiding deep entry into the high-energy second-$\sigma^{*}$ water region.

Specifically, we define the upper cutoff $E_{\mathrm{cut}}$ as the energy corresponding to the first 10% of the integrated area of the main $\sigma^{*}(C-H)$feature (orange vertical line in Figure S9). When the two phases yield noticeably different $E_{\mathrm{cut}}$values within the same model class, the larger value is adopted as the common upper limit for that model. This choice serves two purposes. First, it suppresses most contamination from high-energy second-$\sigma^{*}$ water signals in the $\sigma_{p}^{*}$statistics. Second, it does not eliminate the high-energy tail completely, but retains a limited amount of high-energy signal so that the robustness of the derived trends can still be examined when a modest high-energy contribution is included. In this sense, $E_{\mathrm{cut}}$is not intended to exclude all high-energy states absolutely, but rather to balance two competing requirements: removal of dominant high-energy contamination and retention of a small high-energy buffer for trend-consistency checks.

It is worth noting that the $\sigma^{*}(C-H)$feature behaves differently in cluster and periodic models, reflecting differences in how the two representations encode solvation environments.

- Cluster models (Figure S9a)**.** In both interfacial and bulk-derived clusters, the $\sigma^{*}(C-H)$feature appears as a broad distribution, with only a very subtle relative shift between phases, the interfacial distribution being slightly lower in energy.
- Periodic models (Figure S9b)**.** The interfacial versus bulk contrast becomes much stronger. The interfacial periodic model shows a sharper $\sigma^{*}(C-H)$peak on the high-energy side, whereas the bulk periodic model retains a much broader distribution. This can be rationalized by the semi-immersed interfacial orientation of phenol: in the interfacial model, only the OH-proximal part of phenol is fully solvated, so the corresponding $\sigma^{*}(C-H)$orbitals are broadened, whereas the air-exposed part is only weakly perturbed and retains a sharper, more gas-like character. In bulk, phenol is solvated on all sides; all five $\sigma^{*}(C-H)$orbitals are broadened to varying extents, and because they are not degenerate in energy, these broadened contributions superimpose into a wide composite peak.

The periodic models also show another characteristic feature: in the 9.5–11 eV region, the bulk phase exhibits a higher “background” level than the interface. A useful interpretation is to regard each solvated $\sigma^{*}(C-H)$orbital as contributing a broadened distribution. In bulk, more such distributions contribute, and their low-probability tails overlap in this energy range, thereby increasing the frequency of orbitals exceeding the PDOS-ring–H threshold. By contrast, at the interface, a substantial fraction of the $\sigma^{*}(C-H)$orbitals is not strongly solvated, so tail overlap is reduced and the background level remains lower. At the low-energy onset of the $\sigma^{*}(C-H)$feature (ca. 8–9.5 eV), the two phases show more similar tail behavior, consistent with both being dominated mainly by the low-energy-side tails of broadened distributions.


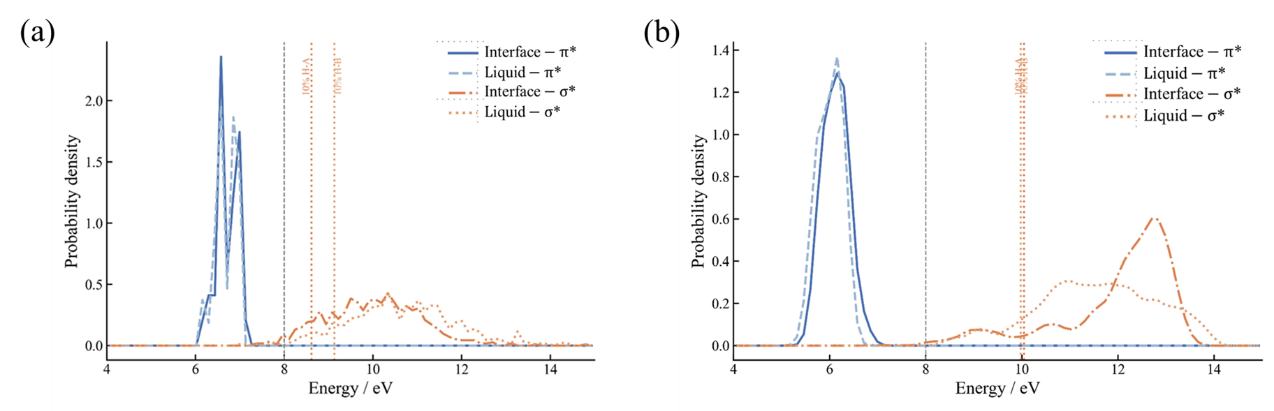


Figure S9. Comparison of π* and σ(C–H)* signatures in cluster vs periodic models and definition of the sampling endpoint. (a) Cluster models; (b) periodic models. The orange dashed line marks the energy corresponding to the first 10% integrated area of the σ(C–H)* feature (sampling endpoint). A: interface; B: bulk.

## S7.4. Final workflow for selecting $\left\{ \boldsymbol{\sigma}_{\boldsymbol{p}}^{\boldsymbol{*}} \right\}$candidates

Based on the above definitions, validations, and contamination analyses, $\left\{ \sigma_{p}^{*} \right\}$ candidates are selected for each configuration using the following workflow.

(1) Broad-capture ranking within the predefined sampling window.

All virtual orbitals within the predefined sampling window are first ranked by PDOS-OH–H, and the top $k$candidates are collected. For cluster models, $k=10$; for periodic models, $k=15$. In practice, however, $k$should be understood as an upper bound rather than as a strictly fixed number for every configuration. If, within a given configuration, the smallest PDOS-OH–H value among the retained candidates is much smaller than the largest one, the lowest-weight tail candidates are removed further. Although no direct one-to-one relationship is assumed between the absolute magnitude of PDOS-OH–H and the ease of electron acceptance, the definition itself implies that orbitals with larger PDOS-OH–H are more likely to participate as effective acceptors in the dark-state electron response. The practical aim is therefore not to preserve a mechanically fixed number of candidates, but to characterize, for each configuration, the $\left\{ \sigma_{p}^{*} \right\}$distribution associated with sufficiently large PDOS-OH–H values.

(2) Rejection of $\pi^{*}$and $\sigma^{*}(C-H)$interferences.

Among these candidates, $\pi^{*}$and $\sigma^{*}(C-H)$interferences are rejected using the PDOS-ring–C and PDOS-ring–H thresholds, respectively. For PDOS-ring–C, the rejection criterion is kept the same for all models and phases: in both cluster and periodic models, and for both interface and liquid phases, any orbital with a PDOS-ring–C value greater than or equal to 0.01 is classified as a $\pi^{*}$interference and removed.

For PDOS-ring–H, the threshold depends on the model type and phase. In the cluster models, the rejection threshold is set to 0.004 for the interfacial phase and 0.003 for the liquid phase. In the periodic models, the threshold is 0.0015 for both phases. Any orbital with a PDOS-ring–H value greater than or equal to the corresponding threshold is classified as a $\sigma^{*}(C-H)$interference and removed.

In addition, because overlap among PDOS-ring–H peaks can generate a “neighborhood contamination” effect, whenever an orbital is flagged by PDOS-ring–H, we additionally remove all orbitals within $\pm0.01$eV of that orbital. This value is identical to the FWHM adopted in the DOS calculations. The rejected orbitals are further checked against their real-space orbital-density distributions to confirm that they indeed exhibit the corresponding $\pi^{*}$or $\sigma^{*}(C-H)$interference character.

(3) Final removal of high-energy second-$\sigma^{*}$ water signals.

Finally, a small number of the remaining orbitals are spot-checked, and those whose morphology is clearly assignable to the second-$\sigma^{*}$ water class (as defined in Figure S8) are removed. This final step ensures that the resulting statistics are dominated by low-energy, phenol-coupled $\sigma_{p}^{*}$ acceptors rather than by dense high-energy water $\sigma^{*}$-like manifolds.

Taken together, this workflow combines broad-capture ranking, fragment-based decontamination, and upper-window/morphology control to define a practically robust $\left\{ \sigma_{p}^{*} \right\}$ensemble for subsequent phase-resolved statistics.

## S7.5. Representative screening examples in minimal models

Table S3. Relative orbital energies and PDOS values of representative virtual orbitals in the phenol–2H₂O model. PDOS-OH–H is scaled by 10³; PDOS-ring–C and PDOS-ring–H are scaled by 10²

| Orb-Number | E(eV) | OH-H | Ring-C | Ring-H |
| --- | --- | --- | --- | --- |
| 37 | 5.86 | **12.22** | 0.17 | 0.02 |
| 38 | 6.13 | 0.49 | 0.03 | 0.05 |
| 39 | 6.20 | 3.83 | **0.30** | 0.03 |
| 40 | 7.57 | 1.05 | 0.05 | **0.14** |


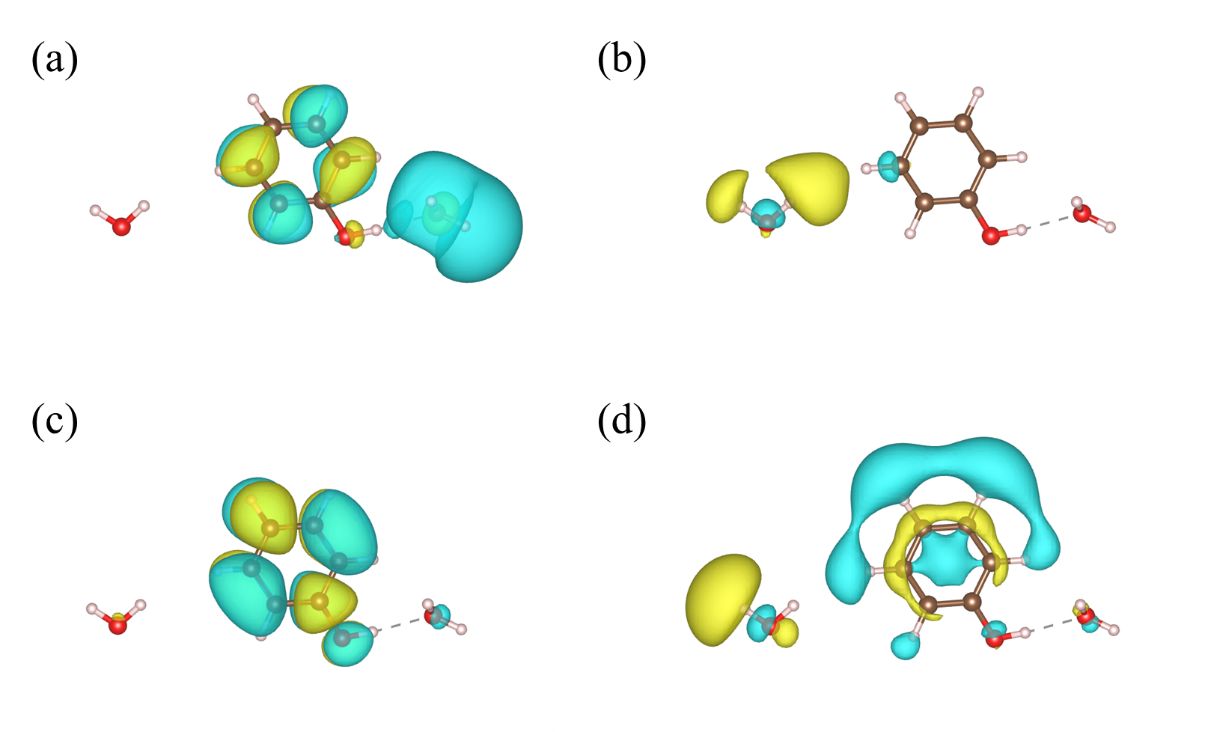


Figure S10. Orbital-density distributions of the representative virtual orbitals listed in Table S3 for the phenol–2H₂O model. Panels (a)–(d) correspond to orbitals 37–40, respectively.

Table S4. Relative orbital energies and PDOS values of representative virtual orbitals in the phenol–6H₂O model. PDOS-OH–H (OH-H) is scaled by 10³; PDOS-ring–C (Ring-C) and PDOS-ring–H (Ring-H) are scaled by 10².

| Orb-Number | E(eV) | OH-H | Ring-C | Ring-H |
| --- | --- | --- | --- | --- |
| 56 | 4.63 | 0.32 | 0.00 | 0.00 |
| 57 | 5.26 | 0.00 | 0.00 | 0.00 |
| 58 | 5.71 | 2.53 | **2.80** | 0.39 |
| 59 | 6.01 | **6.21** | 0.06 | 0.12 |
| 60 | 6.21 | **1.92** | 0.28 | 0.30 |
| 61 | 6.31 | 0.03 | 0.00 | 0.13 |
| 62 | 6.72 | **5.62** | 0.01 | 0.09 |
| 63 | 6.94 | 0.71 | 0.03 | 0.03 |
| 64 | 6.95 | **3.86** | 0.01 | 0.00 |


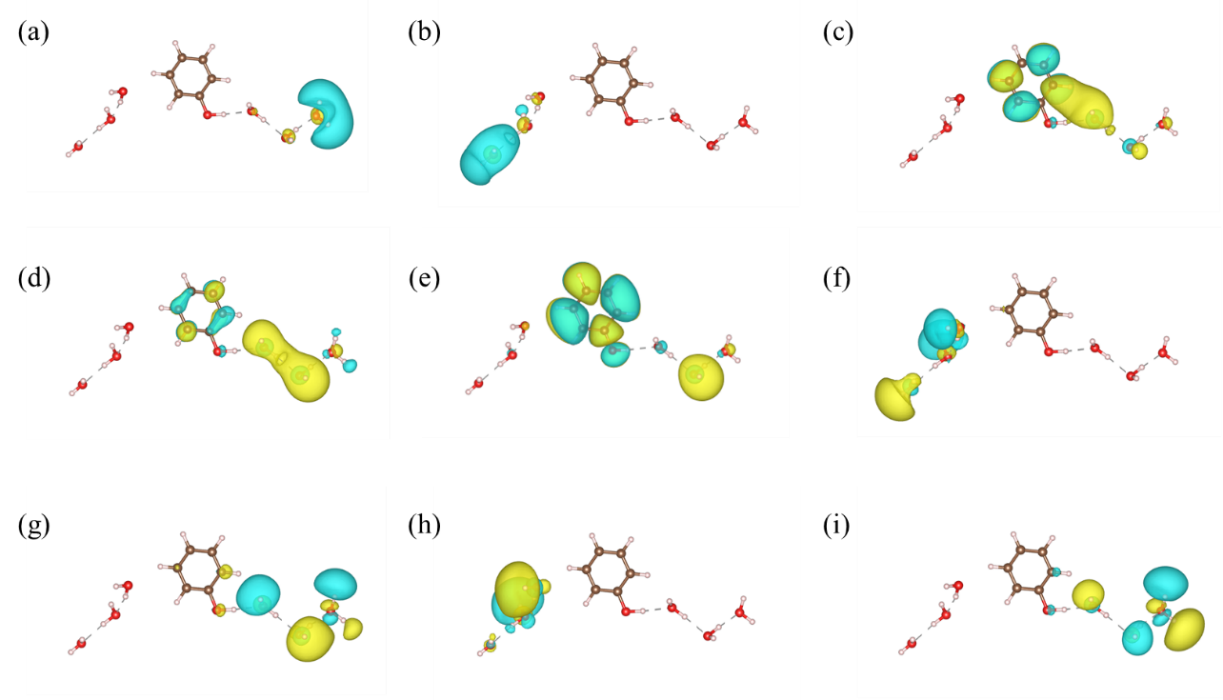


Figure S11. Orbital-density distributions of the representative virtual orbitals listed in Table S4 for the phenol–6H₂O model. Panels (a)–(i) correspond to orbitals 56–64, respectively.

# S8. Density-response probe $\boldsymbol{\Delta}\boldsymbol{\rho}$: definition, orbital matching, and SEAR localization

## S8.1. Rationale for introducing $\boldsymbol{\Delta}\boldsymbol{\rho}$

As discussed in the main text, in order to connect $\sigma_{p}^{*}$energetics to local solvent structure, we need to answer a more specific question for each configuration: where, within a given virtual orbital, the electron associated with the phenolic $\pi\sigma_{p}^{*}$(dark-state) channel preferentially responds.

Inspection of static virtual-orbital isosurfaces at the unperturbed geometry can only show where the orbital density is mainly distributed, i.e., where the electron density of that virtual orbital is concentrated. However, this does not directly reveal which part of that density is actually relevant to the dark-state electron response originating from phenol. In other words, the static orbital map contains the full spatial distribution of the virtual orbital, rather than the incremental component most directly coupled to O–H elongation and to the dark-state electron-transfer direction.

We therefore introduce a finite-displacement density-response probe, $\Delta\rho$, driven by O–H stretching, to extract the electron-density response most sensitive to O–H elongation and to first identify the region of strongest response. This region is then evaluated together with the density distribution of the original, unperturbed orbital to define the solvent-side acceptor region relevant to the dark-state electron response. This combined criterion also excludes cases in which the apparent response occupies only a very small fraction of the original orbital density and therefore does not represent a meaningful dark-state electron-transfer destination. This then provides the basis for subsequent SEAR (solvent-side electron-accumulation region) definition and structural statistics.

## S8.2. Construction of $\boldsymbol{\Delta}\boldsymbol{\rho}$and the choice of $\boldsymbol{\Delta}\boldsymbol{r}$

For each configuration, a finite elongation is imposed along the phenolic O–H bond direction while all other atoms are kept fixed. The spatial distributions (cube files) of the target orbital at the reference and stretched geometries are then exported, and the difference response is constructed as

$$\Delta\rho=\rho_{i}\left( \text{stretched} \right)-\rho_{i}\left( \text{ref} \right) (6)$$

where $\rho_{i}(\text{ref})$is the orbital density of the target orbital at the reference geometry and $\rho_{i}(\text{stretched})$is the density of the corresponding orbital at the stretched geometry.

In the present work, an O–H elongation of $\Delta r=0.10$Å is used. This choice reflects a methodological compromise. If the displacement is too small, the difference signal approaches numerical noise and does not provide a robust basis for identifying a clear response region. If the displacement is increased further and brought closer to the O–H elongation range associated with CI1, the electronic response indeed becomes stronger, but pronounced orbital reorganization, orbital mixing, and orbital-index changes also become more likely, thereby reducing both the interpretability of the difference map and its consistency across configurations. The choice $\Delta r=0.10$Å therefore represents a practical balance: it produces a clear density response while still keeping the orbital character largely trackable and avoiding premature entry into the regime of strong state/orbital reorganization.

It should also be noted that, compared with the original orbital, $\Delta\rho$typically becomes visible only at substantially smaller isovalue thresholds. This is because the subtraction removes the dominant background distribution of the original orbital and retains only the weaker incremental component induced by O–H elongation. Accordingly, $\Delta\rho$ is not intended to redefine the full orbital shape, but rather to extract the localized density change directly associated with the O–H-stretch response.

## S8.3. Orbital matching and phase handling

$\sigma_{p}^{*}$ candidates are selected only at the reference geometry using the PDOS criteria; $\Delta\rho$is used to localize the spatial region relevant to the dark-state electron response, rather than to re-screen acceptor orbitals. Before constructing $\Delta\rho$, it is therefore necessary to determine which orbital at the stretched geometry corresponds to the same target orbital at the reference geometry.

In most cases, the orbital index remains unchanged upon stretching, so the orbital with the same index can be paired directly. However, as noted above, the high-energy region often contains densely packed virtual orbitals with small energy separations, making orbital reorganization, orbital mixing, or orbital-index changes more likely during elongation. In such cases, orbital index alone is no longer sufficient to ensure correct matching, and nearby orbitals must be examined to identify the closest candidate. In practice, the search is first performed within a neighboring range of $\pm1$orbital indices, and extended to $\pm2$only when necessary. If no reliable one-to-one correspondence can be established, or if clear strong mixing is observed such that physical continuity is lost, that orbital is excluded from $\Delta\rho$construction and from the subsequent SEAR-localization analysis.

In addition, the overall molecular-orbital phase is arbitrary. As a result, the two corresponding orbitals before and after elongation may require either direct subtraction or, after phase inversion, an effectively additive treatment. In practice, we first examine whether the orbital phases are consistent. If the phases are consistent, the difference is taken directly; if the phases are opposite, the sign of one orbital is flipped before differencing. This avoids artificial differencing artifacts arising solely from arbitrary orbital-phase choice and ensures that $\Delta\rho$reflects the genuine O–H-stretch-driven electron response rather than a spurious phase mismatch.

## S8.4. SEAR definition and acceptor-site localization

In the present work, the solvent-side electron-accumulation region (SEAR) is assigned from the spatial overlap between the unperturbed orbital density and the positive density response induced by O–H elongation. This definition avoids assigning the acceptor site from the $\Delta\rho$map alone and provides a reproducible procedure for identifying the solvent-side region most directly associated with the orbital response.

For each candidate $\sigma_{p}^{*}$orbital, the unperturbed orbital wavefunction was first converted into an orbital-density field,

$$\rho_{\mathrm{orb}}\left( \mathbf{r} \right)=\mid\psi_{\sigma_{p}^{*}}\left( \mathbf{r} \right)\mid^{2}\ldots\ldots\ldots\ldots\ldots\ldots\ldots\ldots\ldots\ldots\ldots\ldots\ldots\ldots\ldots\ldots\ldots\ldots\ldots\ldots\ldots\ldots\ldots\ldots\ldots\ldots(7)$$

Only the electron-accumulation component of the O–H-elongation-induced density difference was retained,

$$\Delta\rho_{+}\left( \mathbf{r} \right)=\max\left[ \Delta\rho\left( \mathbf{r} \right),0 \right]\ldots\ldots\ldots\ldots\ldots\ldots\ldots\ldots\ldots\ldots\ldots\ldots\ldots\ldots\ldots\ldots\ldots\ldots\ldots\ldots\ldots\ldots\ldots\ldots(8)$$

because SEAR refers to the solvent-side electron-accumulation region rather than the electron-depletion region. Both $\rho_{\mathrm{orb}}(\mathbf{r})$and $\Delta\rho_{+}(\mathbf{r})$were then normalized by their volume integrals to reduce the influence of differences in absolute density magnitude among different orbitals and density-difference files.

A normalized spatial overlap field was constructed on the common three-dimensional grid as

$$\Omega\left( \mathbf{r} \right)=\sqrt{\tilde{\rho}_{\mathrm{orb}}\left( \mathbf{r} \right)\tilde{\Delta\rho}_{+}\left( \mathbf{r} \right)}\ldots\ldots\ldots\ldots\ldots\ldots\ldots\ldots\ldots\ldots\ldots\ldots\ldots\ldots\ldots\ldots\ldots\ldots\ldots\ldots\ldots\ldots\ldots\ldots.(9)$$

where $\tilde{\rho}_{\mathrm{orb}}$and $\tilde{\Delta\rho}_{+}$denote the normalized orbital density and normalized positive density response, respectively. In this form, SEAR assignment is based on the voxel-level spatial correspondence between the original orbital density and the positive response, rather than on visual inspection of either distribution alone.

In practice, all voxels were ranked by their overlap contribution, $\Omega(\mathbf{r})dV$. Voxels with the largest cumulative overlap contribution were retained and subjected to three-dimensional connected-component analysis, in which spatially continuous voxels were grouped into individual overlap regions. The connected component with the largest overlap contribution was defined as the SEAR for the corresponding orbital and was used in the subsequent $N$and $P$analyses.

This voxel-overlap procedure provides a consistent operational definition of SEAR across orbitals with different spatial extents and response magnitudes. Representative applications of this procedure to complex truncated-cluster environments are shown in Figures S15 and S16.

In both cases, the unperturbed orbital density and the O–H-elongation-induced $\Delta\rho$map exhibit a discernible spatial overlap, indicating that the solvent-side response identified by $\Delta\rho$remains connected to the original virtual-orbital density even in denser multi-water environments (Figure S15a,b; Figure S16a,b). Compared with minimal phenol–water clusters, the background orbital density is more complex in these truncated clusters, making visual assignment of the overlap region less straightforward. The voxel-overlap procedure resolves this ambiguity by automatically extracting the dominant connected overlap region, which is consistent with the target region indicated by the orbital-density/$\Delta\rho$ correspondence (Figure S15d; Figure S16d). These examples support the reproducibility of the SEAR definition and its applicability to realistic multi-water solvent environments.

## S8.5. Representative $\boldsymbol{\Delta}\boldsymbol{\rho}$maps in minimal and cluster models

Representative $\Delta\rho$maps are shown for both minimal and cluster models to illustrate the applicability of the method across systems of different complexity; the corresponding physical discussion and interpretation are given in the main text.


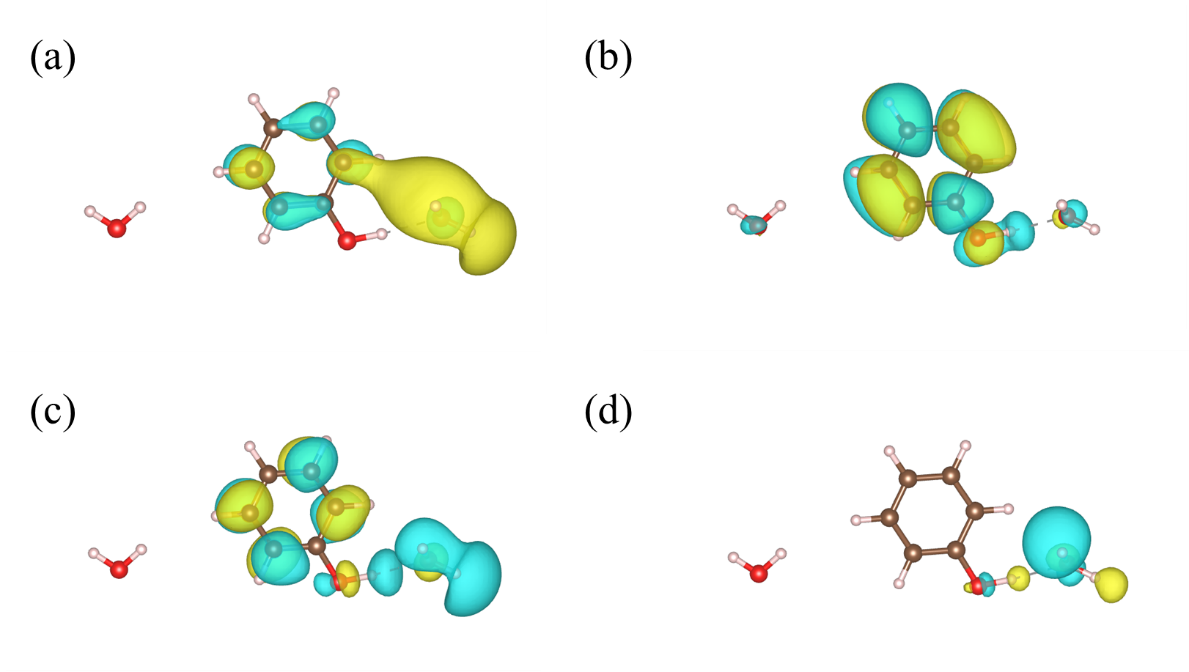


Figure S12. Electron-density-difference maps (Δρ) of the representative virtual orbitals listed in Table S3 for the phenol–2H₂O model after O–H stretch perturbation.


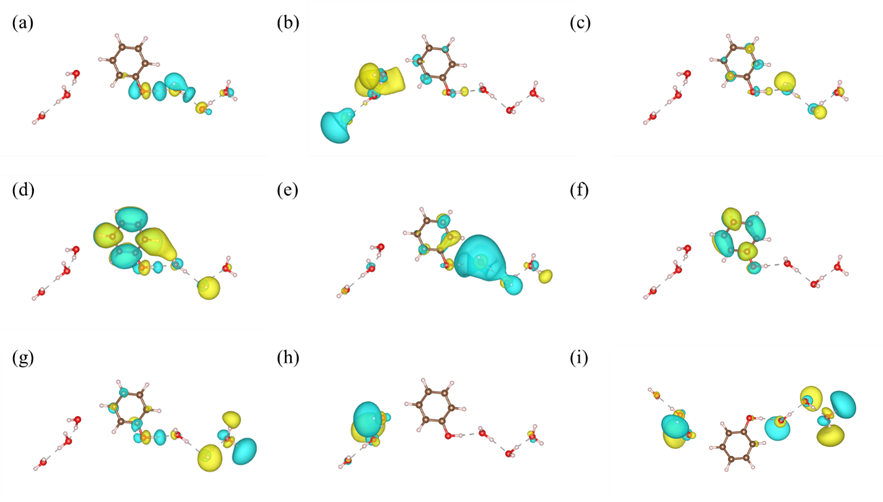


Figure S13. Electron-density-difference maps (Δρ) of the representative virtual orbitals listed in Table S4 for the phenol–6H₂O model after O–H stretch perturbation.


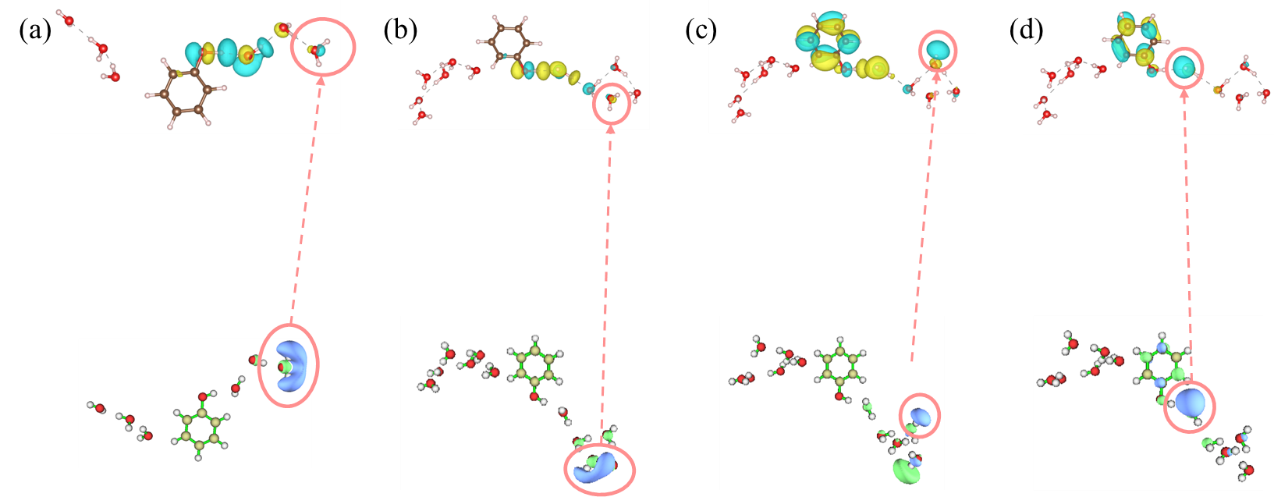


Figure S14**.** Orbital–SEAR overlap and its correspondence with PDOS magnitude in representative cluster microenvironments. Panel (a) shows a case with almost no spatial overlap, whereas panels (b)–(d) show the same qualitative trend in more complex environments, with PDOS-OH–H values of 0.16, 2.08, and 9.38, respectively. **
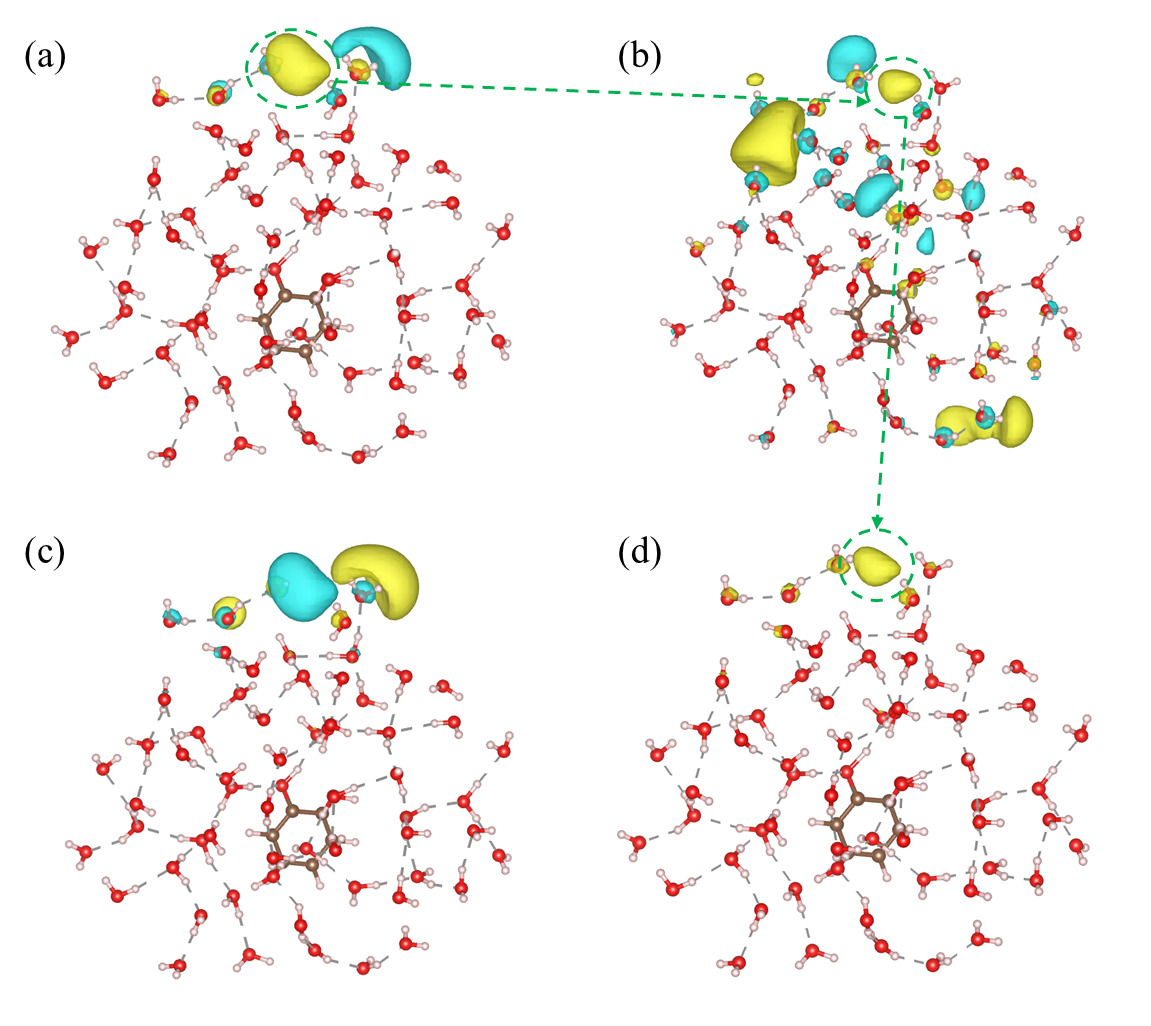
**

Figure S15. (a) Unperturbed orbital-density distribution of the selected candidate $\sigma_{p}^{*}$orbital. (b) O–H-elongation-induced density difference $\Delta\rho$, showing the electron-accumulation response. (c) Orbital-density distribution after O–H elongation. (d) SEAR region automatically identified by the voxel-based orbital–response overlap procedure.

**
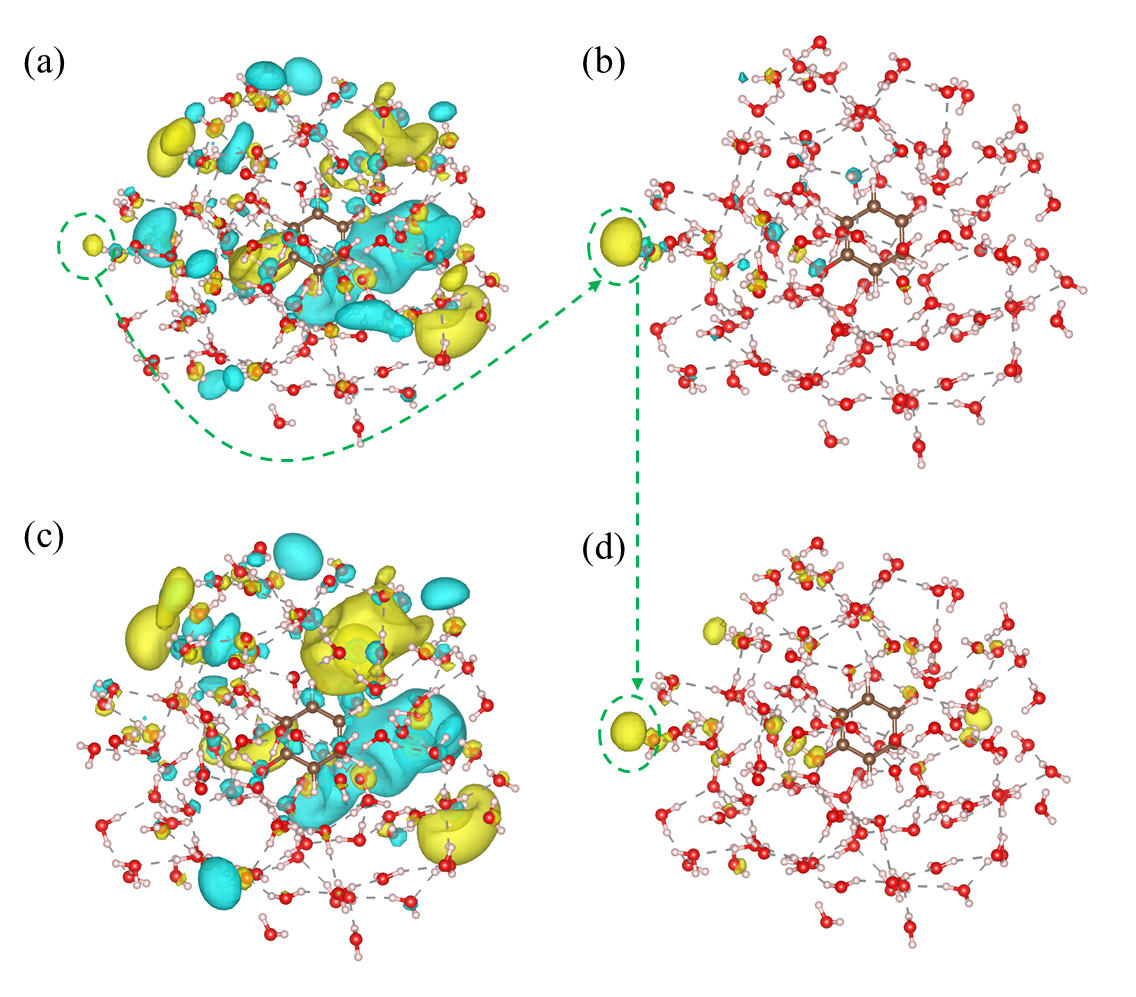
**

Figure S16. (a) Unperturbed orbital-density distribution of the selected candidate $\sigma_{p}^{*}$orbital. (b) O–H-elongation-induced density difference $\Delta\rho$, showing the electron-accumulation response. (c) Orbital-density distribution after O–H elongation. (d) SEAR region automatically identified by the voxel-based orbital–response overlap procedure.

# S9. Motif classification and local descriptors

Because thermal fluctuations in MD configurations are substantial and instantaneous hydrogen-bond geometries are often distorted, a relatively loose geometric hydrogen-bond criterion, suitable for MD configurations, was adopted in the present work. Specifically, a hydrogen bond was counted when a donor–acceptor pair satisfied $d_{O\cdots O}\leq3.5$Å and $\angle H-O\cdots O\leq{30}^{\circ}$, equivalently $\angle O-H\cdots O\geq{150}^{\circ}$. All analyses that rely on hydrogen-bond assignment, including local water-motif classification and the identification of hydrogen-bond-saturated or unsaturated water oxygens, were based on this unified criterion.

In contrast to this hard hydrogen-bond definition, the SEAR-centered descriptor $P$was defined as a continuous soft geometric descriptor to characterize the local directional pressing around the SEAR-associated dangling H direction. Within the SEAR-centered directional region, water oxygen atoms accepting fewer than two hydrogen bonds were first identified as unsaturated acceptor oxygens. For each such oxygen, the $d_{H\cdots O}$distance to the target SEAR-associated H atom was mapped to a distance-dependent weight: the contribution was set to 1 for $d_{H\cdots O}\leq2.0$Å, to 0 for $d_{H\cdots O}\geq4.0$Å, and linearly interpolated between 2.0 and 4.0 Å. Thus, $P$is not a binary hydrogen-bond count, but a continuous measure of the degree of local approach, and hence the potential directional influence, of unsaturated acceptor oxygens around the SEAR.

The directional region for $P$was defined as a cone centered along the SEAR-associated O–H direction, with the cone angle $\theta$measured between the O–H bond direction and the vector from the target H atom to a candidate unsaturated acceptor oxygen (Figure S17). The 2.0–4.0 Å distance-decay window and the ${60}^{\circ}$cone were chosen to balance coverage and specificity: a narrower distance or angular range would mainly retain near-ideal hydrogen-bond-like contacts and miss distorted but relevant local constraints, whereas a broader range would introduce lateral or second-shell oxygens and dilute the first-shell directional signal. Therefore, $P$was designed to represent a first-solvation-shell, direction-sensitive local pressure imposed by unsaturated acceptor oxygens around the SEAR-associated H direction. The sensitivity of this geometric definition is further examined in Section S10.


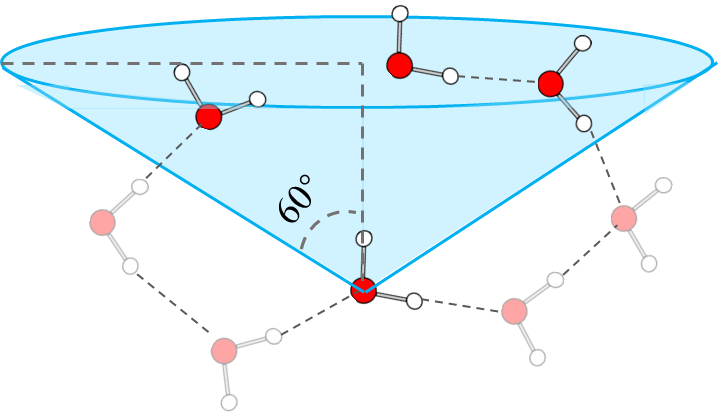


Figure S17. Schematic definition of the SEAR-centered directional region used for the $P$ descriptor.

**
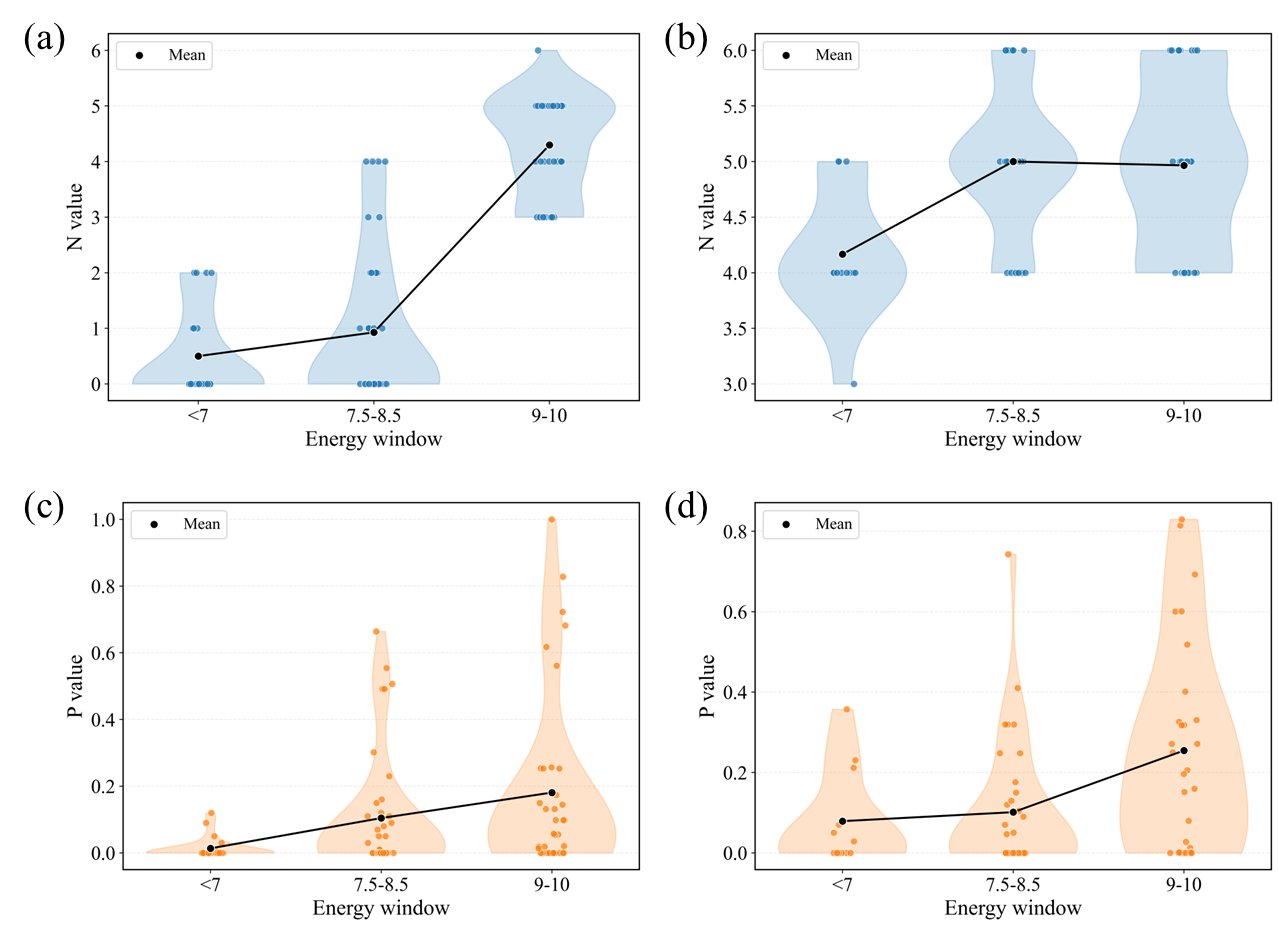
**

Figure S18. Distributions of the SEAR-centered descriptors $N$and $P$in the interfacial and bulk periodic models. Panels (a,c) show the interfacial results and panels (b,d) show the bulk results; panels (a,b) correspond to $N$, and panels (c,d) to $P$.

# S10. Robustness and sensitivity analysis of operational definitions

## S10.1. Rationale and validation of the upper sampling cutoff E_cut_

The upper sampling cutoff $E_{\mathrm{cut}}$ was used to define the low-energy virtual-orbital window for identifying the solvent-side $\sigma_{p}^{*}$acceptor manifold. This window was set before the dense high-energy virtual-orbital region, where diffuse water-$\sigma^{*}$ components, C-H* mixing, and perturbation-induced orbital reorganization become more pronounced.

In the present analysis, the adopted cutoff includes the low-energy solvent-side acceptor states relevant to SEAR assignment. Extending the window to higher energies would introduce additional virtual orbitals whose density responses are not consistently associated with the targeted $\sigma_{p}^{*}$-type solvent-side electron-accommodation channel. Such orbitals are therefore not included in the same sampling window.

Representative high-energy orbitals in phenol–2H_2_O and phenol–6H_2_O models were examined to evaluate the behavior of orbitals beyond this window. In the high-energy region, a large PDOS-OH-H value does not necessarily correspond to a physically meaningful solvent-side $\sigma_{p}^{*}$acceptor state. For example, in the phenol–2 H_2_O model, orbital 41 has a PDOS-OH–H value of 3.86, larger than that of several lower-energy orbitals, including orbital 38 with a PDOS-OH–H value of 0.49. However, its O–H-elongation-induced $\Delta\rho$response shows little effective spatial overlap with the corresponding unperturbed orbital-density distribution (Figure S19a,c), suggesting that this large PDOS-OH–H contribution may mainly arise from diffuse high-energy water-$\sigma^{*}$ or CH*-mixed character.

High-energy orbitals also show stronger sensitivity to perturbation-induced reorganization. Because adjacent virtual orbitals are closely spaced, O–H elongation can redistribute the original orbital character over neighboring orbitals. For instance, orbital 64 before O–H elongation is partially redistributed into two adjacent orbital components after perturbation (Figure S19d–f). In this case, a one-to-one density-difference construction no longer provides a clean local electron-response descriptor.

These observations support the use of $E_{\mathrm{cut}}$as a practical upper boundary for the low-energy $\sigma_{p}^{*}$ acceptor sampling window. The selected cutoff retains the orbitals used for the present SEAR analysis while avoiding high-energy states with inconsistent orbital-density and $\Delta\rho$correspondence.

**
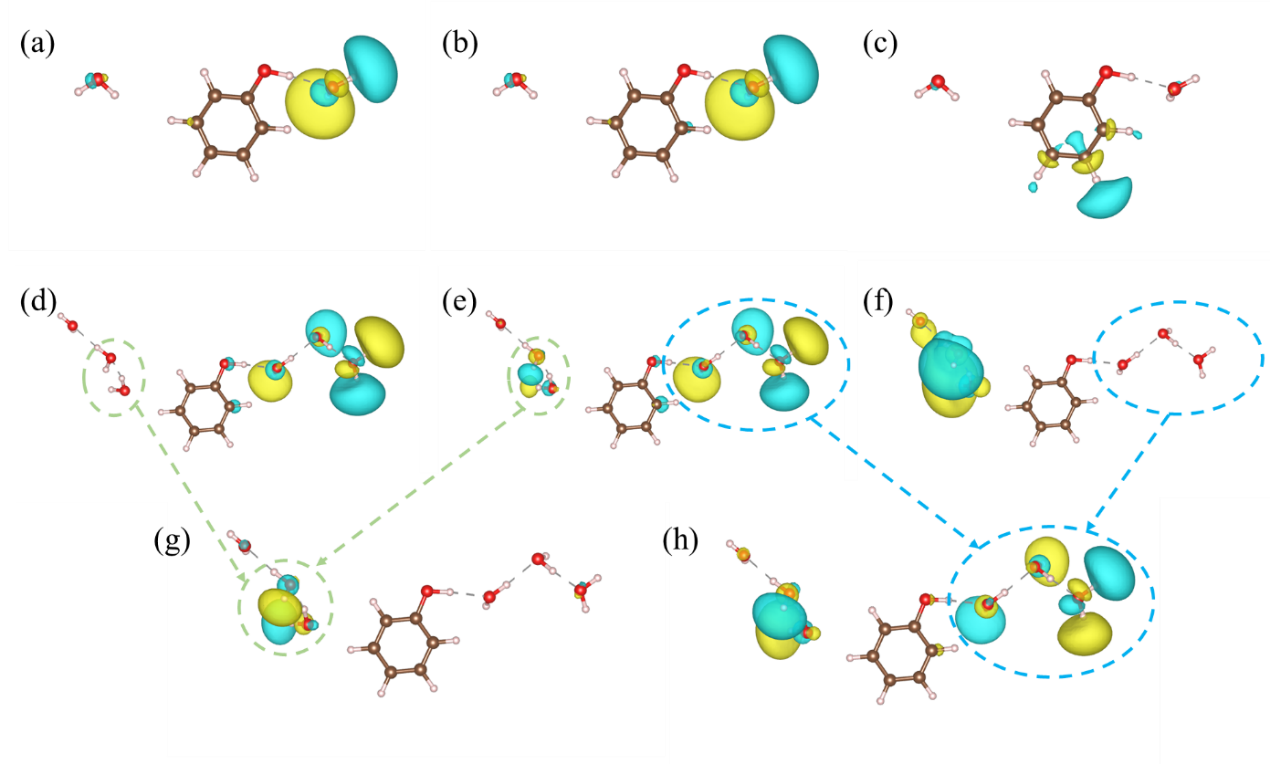
**

Figure S19 (a–c) Unperturbed orbital-density distribution, O–H-elongated orbital-density distribution, and the corresponding $\Delta\rho$map for molecular orbital 41 in the phenol–2H_2_O model. (d–f) Orbital-density distributions of orbital 63 after O–H elongation, orbital 64 before O–H elongation, and orbital 64 after O–H elongation in the phenol-6H_2_O model, respectively. (g,h) Corresponding $\Delta\rho$maps obtained from the pairs shown in (d,e) and (f,e), respectively.

## S10.2. Sensitivity to the PDOS-OH–H ranking depth k

The PDOS-OH–H ranking depth $k$was used to define an initial candidate pool of solvent-side acceptor orbitals in configurations with different water counts and local hydrogen-bond topologies. Because a fixed absolute PDOS-OH–H threshold is not readily transferable across different snapshots, the top-$k$ virtual orbitals ranked by PDOS-OH–H were first retained within each configuration. This initial pool was then refined by the PDOS-ring–C and PDOS-ring–H filters, the upper sampling cutoff $E_{\mathrm{cut}}$, real-space morphology inspection, and $\Delta\rho$-based SEAR localization.

In this workflow, $k$controls only the breadth of the initial candidate pool and does not define the final $\sigma_{p}^{*}$acceptor manifold. A larger $k$increases the probability of capturing relevant solvent-side acceptor orbitals but also introduces more low-PDOS-OH–H tail states, whereas an overly small $k$may remove relevant candidates before the subsequent filtering steps.

In practice, $k$was chosen by comparing the lowest PDOS-OH–H value in the retained top-$k$ set with the high-ranking PDOS-OH–H values from the same configuration. The third-largest PDOS-OH–H value was used as an internal reference, since the highest-ranked orbitals can occasionally include $\pi^{*}$or CH* contamination before rejection filters are applied. As shown in Figure S20, $k=10$ captures the main candidate orbitals in the cluster models, whereas $k=15$ provides a broader initial window for the periodic models, where the number of waters and the virtual-orbital density are larger.


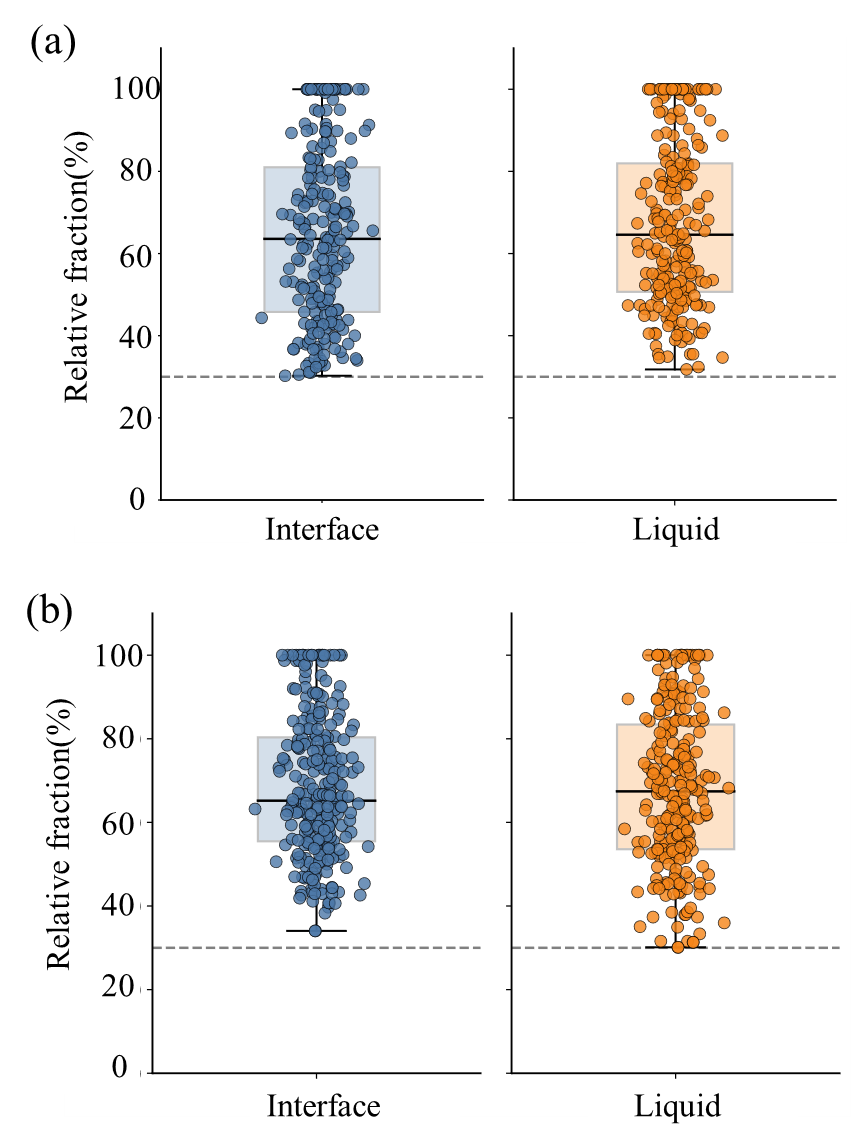


Figure S20 (a) Distribution of the difference between the lowest PDOS-OH–H value retained in the top-$k$ candidate pool and the reference PDOS-OH–H value for the interfacial and bulk-like cluster models with $k=10$. (b) Corresponding distribution for the periodic interfacial and bulk models with $k=15$. The reference value is defined as the third-largest PDOS-OH–H value in each configuration.

To evaluate ranking-depth sensitivity, the periodic-model analysis was repeated with $k=12$, 15, and 18. As shown in Figure S21, the interfacial $\varepsilon\left( \sigma_{p}^{*} \right)$distribution remains lower in energy and broader than the bulk distribution for all three choices. At the 75% quantile, the bulk distribution is higher than the interfacial distribution by approximately 0.72 eV for $k=12$, 0.70 eV for $k=15$, and 0.60 eV for $k=18$. Thus, the phase-resolved separation is retained over the tested ranking-depth range.

Changes in the distributions with increasing $k$further indicate which orbital populations are introduced as the candidate pool is broadened. As $k$increases, the low-energy interfacial peak decreases in relative intensity, while the high-energy contribution becomes more pronounced. This suggests that the low-energy interfacial $\sigma_{p}^{*}$ acceptor orbitals generally have relatively large PDOS-OH–H values and are already captured at smaller $k$, whereas the additional lower-PDOS-OH–H tail orbitals mainly contribute to higher-energy regions. The bulk distribution shows a weaker dependence on $k$, with only minor changes near the low-energy shoulder.

Overall, the main phase-resolved trend is insensitive to reasonable variations in $k$. The interfacial ensemble consistently shows a lower-energy and broader $\varepsilon\left( \sigma_{p}^{*} \right)$distribution than the bulk ensemble, supporting the use of $k=15$as the initial ranking depth for the periodic analysis.


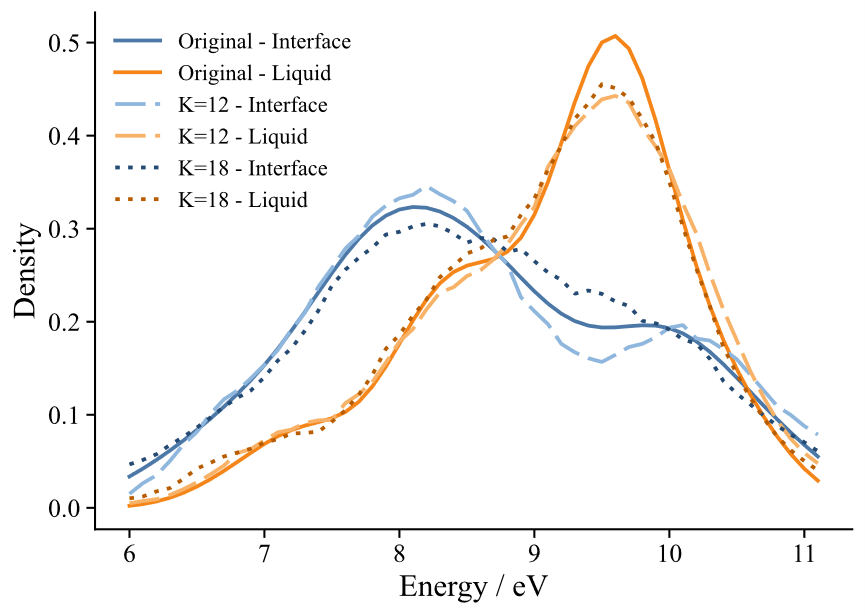


Figure S21 Phase-resolved $\varepsilon(\sigma_{p}^{*})$distributions obtained from the periodic models using $k=12$, 15, and 18.

## S10.3. Rationale and sensitivity of the SEAR-centered geometry used for P

As defined in Section S9, $P$quantifies the directional pressing exerted by unsaturated acceptor oxygens around the SEAR-associated H direction. The standard definition uses a 2.0–4.0 Å distance-decay window and a ${60}^{\circ}$conical region to retain directionally relevant first-shell contacts while limiting contributions from lateral or second-shell oxygens. To evaluate the sensitivity of this geometry, $P$was recalculated with alternative cone angles.

A suitable conical region should sample the environment in front of the SEAR-associated H atom rather than the general abundance of unsaturated oxygens in a wider solvation region. A very narrow cone may exclude distorted but still relevant first-shell arrangements, whereas an overly broad cone includes lateral and second-shell oxygens that no longer represent direct directional pressing on the target H direction.

Figure S22 summarizes the resulting sensitivity test. Broadening the cone increases the absolute $P$values across the $\varepsilon\left( \sigma_{p}^{*} \right)$windows because more unsaturated oxygens are included. However, the energy-dependent variation becomes weaker and can be nearly removed, indicating that the broadened geometry mixes the intended first-shell directional signal with more remote, less specific contributions. Since the low-energy-region differences are relatively subtle, such overinclusive angular sampling can obscure the variation that $P$is intended to describe.

Using a ${60}^{\circ}$cone preserves a practical balance between coverage and directionality. Moderately distorted first-shell contacts remain included, while lateral and second-shell contributions are limited. Under this definition, lower $\varepsilon\left( \sigma_{p}^{*} \right)$ windows remain associated with weaker local pressing, whereas higher-energy windows correspond to more crowded SEAR-centered environments. Thus, the observed structure–energy relationship is retained within a physically meaningful first-shell directional region rather than arising from an arbitrary cone choice.


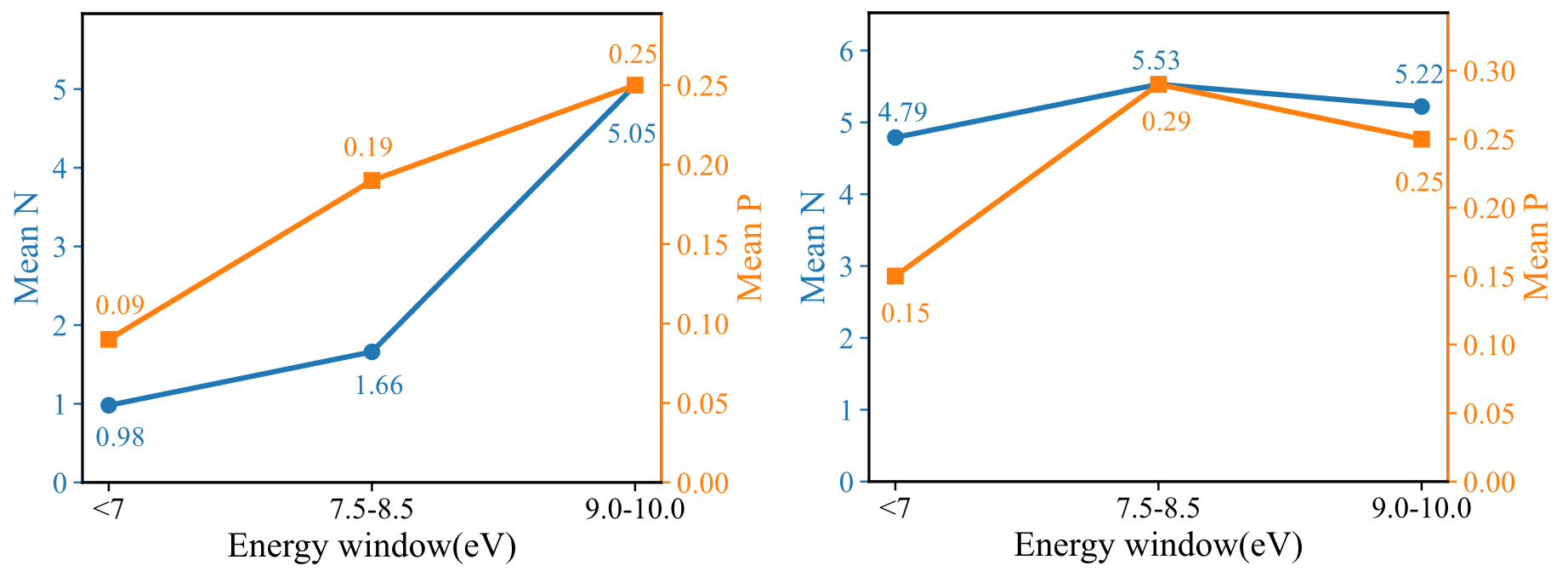


Figure S22 (a) Variations of $N$and $P$with cone angle for the interfacial cluster models. (b) Corresponding variations for the bulk-like cluster models. The comparison evaluates how broadening the conical region affects the local-environment descriptors across different $\varepsilon(\sigma_{p}^{*})$windows.

## S10.4. Statistical robustness and representativeness of the periodic snapshots

The periodic-model analysis was based on 20 snapshots for each phase. To examine whether this sample size is sufficient for supporting the phase-resolved $\varepsilon\left( \sigma_{p}^{*} \right)$trend, we performed a reduced-sample test and a snapshot-level bootstrap analysis.

In the reduced-sample test, the $\varepsilon\left( \sigma_{p}^{*} \right)$distributions were recalculated using only 10 snapshots per phase. As shown in Figure S23a, reducing the sample size changes some local distributional details and increases the uncertainty range, but the main trend remains unchanged: the interfacial distribution is still lower in energy and broader than the bulk distribution. The bulk–interface mean separation is approximately 0.8 eV within the leading 75% cumulative-density region and approximately 0.6 eV over the full distribution, both consistent with the 20-snapshot result.

For the bootstrap analysis, each snapshot was treated as one independent resampling unit. The mean $\varepsilon(\sigma_{p}^{*})$ of the retained $\sigma_{p}^{*}$ orbitals was first calculated for each snapshot, and bootstrap resampling was then performed over the 20 snapshots with replacement. This avoids treating multiple orbitals from the same snapshot as fully independent samples. As shown in Figure S23b, the full-window observed shift is 0.55 eV, the bootstrap mean is approximately 0.55 eV, and the 95% confidence interval is 0.42–0.69 eV. The confidence interval does not cross zero, indicating that the bulk-upshift relative to the interfacial phase is statistically stable.

This difference from the $\sim0.7$eV value discussed in the main text arises from the different averaging protocols used in the two analyses. The main-text value corresponds to the density-weighted mean shift within the leading 75% cumulative-density region, whereas the bootstrap analysis uses snapshot-level means over the full retained energy window. Direct averaging over the full window gives a bulk–interface difference of approximately 0.49 eV, consistent with the bootstrap estimate. Thus, although the absolute value varies moderately with the statistical definition, all analyses support a clear phase-resolved separation in $\varepsilon(\sigma_{p}^{*})$.

The number of retained $\sigma_{p}^{*}$orbitals and assigned SEARs per snapshot is summarized in Figure S23c,d. Most snapshots retain approximately 7–10 $\sigma_{p}^{*}$orbitals and 5–10 SEAR regions, indicating that the statistics are not dominated by a few exceptional configurations.

Because the $\varepsilon(\sigma_{p}^{*})$ trend is closely linked to the X-1 motif and its forward environment, representativeness of the selected 20 snapshots was further assessed using X-1-related local-environment descriptors. Figure S24 compares the selected 20 snapshots with the last 3000 AIMD frames. The selected snapshots reproduce the main features of the longer trajectory: for the number of water oxygens in the X-1 forward cone, the interfacial phase is more populated in the less crowded region, whereas the bulk phase contributes more strongly in the crowded region; for the X-1 dangling-H pressing distance, the selected snapshots and the full trajectory show consistent phase-dependent distributions. These comparisons indicate that the selected periodic snapshots preserve the average background abundance of the key X-1 local environments and are representative for the subsequent phase-resolved structure–energy analysis.


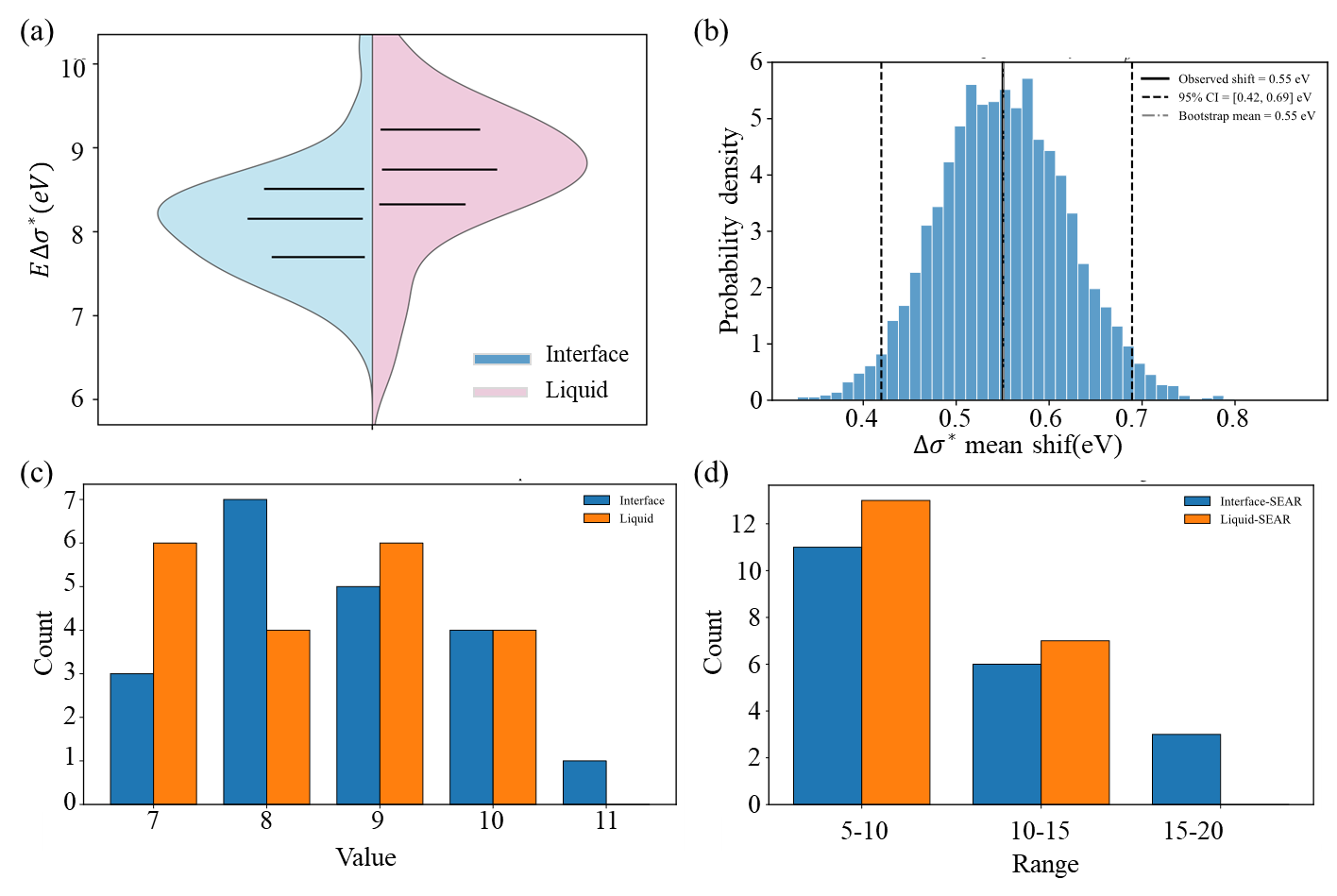


Figure S23 (a) Phase-resolved $\varepsilon(\sigma_{p}^{*})$distributions recalculated using 10 snapshots per phase. (b) Snapshot-level bootstrap distribution of the bulk–interface mean shift, using each periodic snapshot as one resampling unit. (c) Number of retained $\sigma_{p}^{*}$candidate orbitals in each periodic snapshot. (d) Number of assigned SEAR regions in each periodic snapshot.


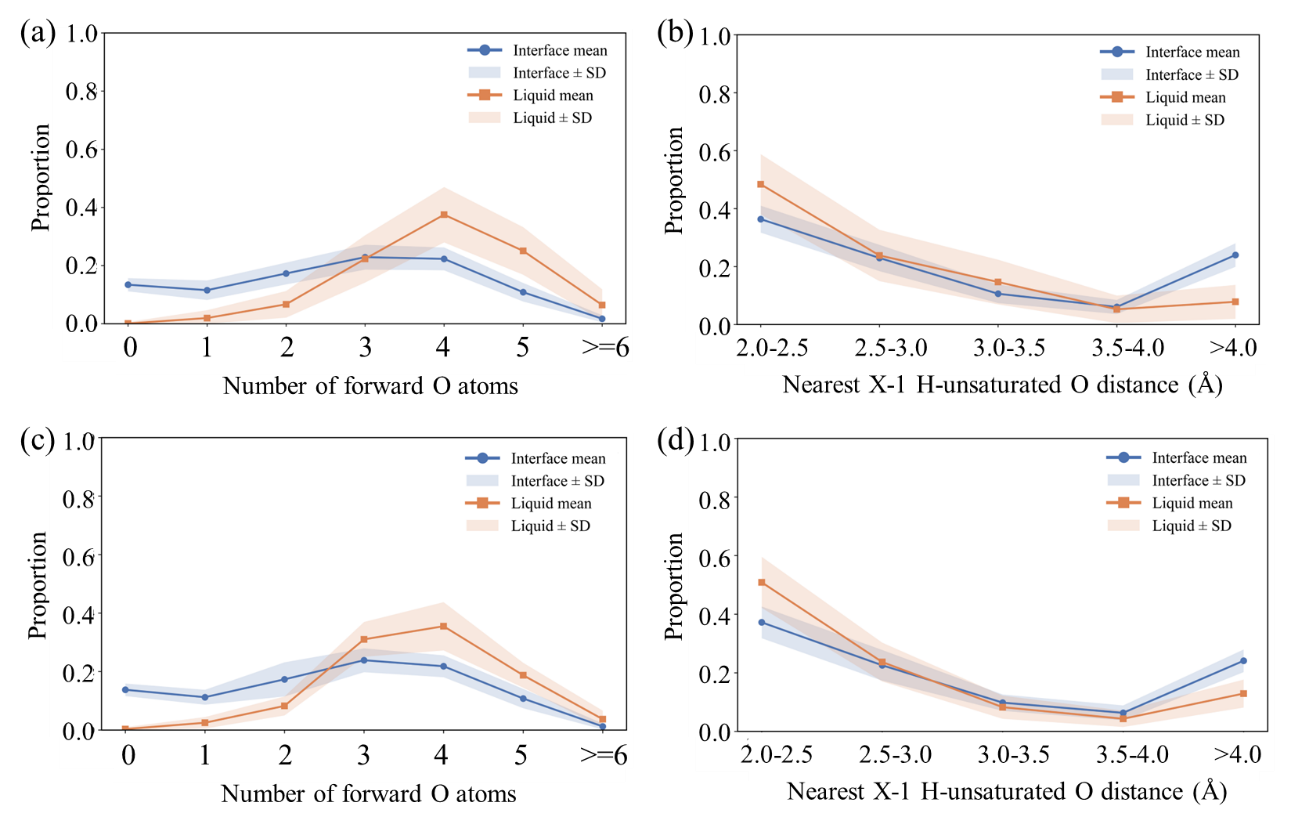


Figure S24 (a,b) Background distributions of the number of forward oxygen atoms and the X-1 dangling-H···O distance to unsaturated acceptor oxygens in the selected 20 periodic snapshots. (c,d) Corresponding distributions obtained from the last 3000 frames of the AIMD trajectories. The comparison evaluates whether the selected snapshots preserve the average X-1 local-environment features sampled in the longer trajectories.

To evaluate whether the 30 MD-extracted cluster snapshots used in the cluster-level analysis are representative of the underlying classical MD trajectories, we compared their water-motif background abundances with those obtained from a larger trajectory-sampled dataset. Specifically, 2000 frames were evenly selected from the last 10,000 MD frames, and for each sampled frame the local environment was reconstructed using the same phenol-centered cutoff strategy as used for cluster extraction.

As shown in Figure S25, the motif fractions obtained from the selected 30 cluster snapshots (Figure S25a,b) are in good agreement with those obtained from the 2000 trajectory-sampled local environments (Figure S25c,d) for both the interfacial and liquid phases. This agreement indicates that the 30 selected clusters capture the average motif-abundance pattern of the corresponding MD trajectories and are therefore representative for the cluster-level statistical analysis. In both datasets, a clear inner–outer contrast is observed: the inner region is dominated by more hydrogen-bond-saturated X-2 motifs, whereas the outer region is enriched in X-1 motifs. The relative fractions of the major motif classes differ only weakly between the 30-cluster subset and the larger trajectory-sampled dataset, further supporting the stability of this motif-distribution pattern.

Importantly, the same outer-region enrichment of X-1 motifs appears in both phases. This result indicates that the finite-cluster boundary creates a similar under-coordinated outer-shell environment in the interfacial and liquid cluster models. Consequently, although the selected 30 clusters are representative of the cluster-extraction protocol, the truncation-generated boundary region partially homogenizes the motif background between the two phases. This provides additional support for the interpretation that finite-cluster truncation can weaken the intrinsic phase contrast and bias structure–energy analyses toward boundary-dominated microenvironments.


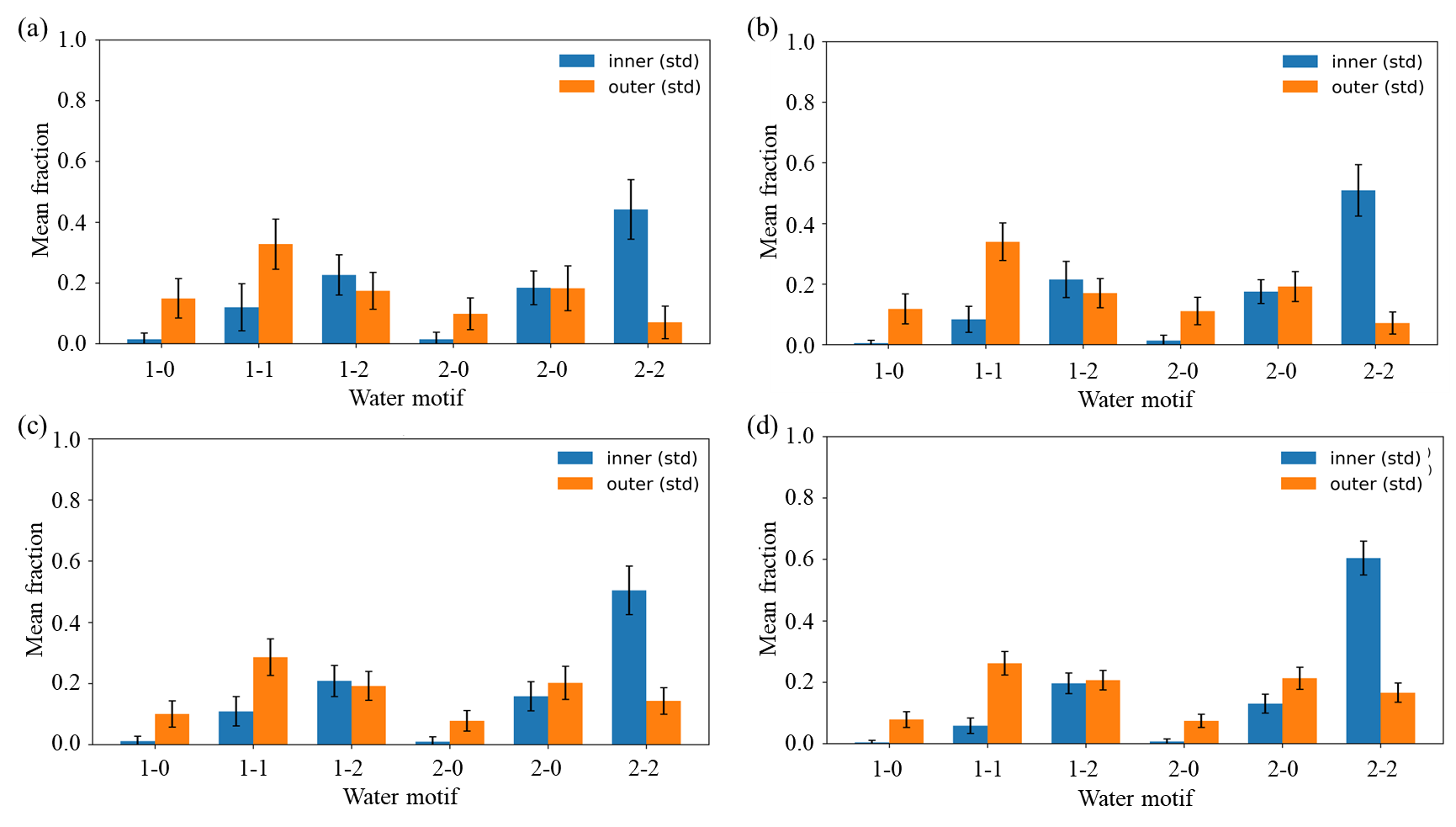


Figure S25 Water-motif background abundances in extracted clusters and trajectory-sampled local environments. (a,b) Mean motif fractions in the extracted interfacial (a) and liquid (b) clusters. (c,d) Mean motif fractions obtained from trajectory-sampled local environments for the interfacial (c) and liquid (d) phases.

# References

[1] M. J. Abraham, T. Murtola, R. Schulz, S. Páll, J. C. Smith, B. Hess, E. Lindahl, *SoftwareX* **2015**, *1-2*, 19.

[2] M. Schauperl, P. S. Nerenberg, H. Jang, L.-P. Wang, C. I. Bayly, D. L. Mobley, M. K. Gilson, *Communications Chemistry* **2020**, *3*, 44.

[3] G. Bussi, D. Donadio, M. Parrinello, *The Journal of Chemical Physics* **2007**, *126*.

[4] M. Parrinello, A. Rahman, *Journal of Applied Physics* **1981**, *52*, 7182.

[5] Y. Nagata, R. E. Pool, E. H. G. Backus, M. Bonn, *Physical Review Letters* **2012**, *109*, 226101.

[6] a) R. Kusaka, S. Nihonyanagi, T. Tahara, *Nature Chemistry* **2021**, *13*, 306; b) R. Kusaka, T. Ishiyama, S. Nihonyanagi, A. Morita, T. Tahara, *Physical Chemistry Chemical Physics* **2018**, *20*, 3002.

[7] T. D. Kühne, T. A. Pascal, E. Kaxiras, Y. Jung, *J Phys Chem Lett* **2011**, *2*, 105.

[8] Y.-i. Yamamoto, Y.-I. Suzuki, G. Tomasello, T. Horio, S. Karashima, R. Mitríc, T. Suzuki, *Physical Review Letters* **2014**, *112*, 187603.

[9] T. Ishiyama, T. Tahara, A. Morita, *Journal of the American Chemical Society* **2022**, *144*, 6321.

[10] A. L. Sobolewski, W. Domcke, *The Journal of Physical Chemistry A* **2001**, *105*, 9275.
